# The Imbalance of p53–Park7 Signaling Axis Induces Iron Homeostasis Dysfunction in Doxorubicin‐Challenged Cardiomyocytes

**DOI:** 10.1002/advs.202206007

**Published:** 2023-03-26

**Authors:** Jianan Pan, Weiyao Xiong, Alian Zhang, Hui Zhang, Hao Lin, Lin Gao, Jiahan Ke, Shuying Huang, Junfeng Zhang, Jun Gu, Alex Chia Yu Chang, Changqian Wang

**Affiliations:** ^1^ Department of Cardiology Shanghai Ninth People's Hospital Shanghai JiaoTong University school of Medicine Shanghai 200001 P. R. China; ^2^ Department of Shanghai Institute of Precision Medicine Shanghai Ninth People's Hospital Shanghai JiaoTong University school of Medicine Shanghai 200135 P. R. China; ^3^ Department of Echocardiography Zhongshan Hospital Fudan University Shanghai 200030 P. R. China

**Keywords:** doxorubicin‐induced cardiotoxicity, ferroptosis, Fe—S cluster, iron homeostasis, p53–Park7 signaling axis

## Abstract

Doxorubicin (DOX)‐induced cardiotoxicity (DoIC) is a major side effect for cancer patients. Recently, ferroptosis, triggered by iron overload, is demonstrated to play a role in DoIC. How iron homeostasis is dysregulated in DoIC remains to be elucidated. Here, the authors demonstrate that DOX challenge exhibits reduced contractile function and induction of ferroptosis‐related phenotype in cardiomyocytes, evidenced by iron overload, lipid peroxide accumulation, and mitochondrial dysfunction. Compared to Ferric ammonium citrate (FAC) induced secondary iron overload, DOX‐challenged cardiomyocytes show a dysfunction of iron homeostasis, with decreased cytoplasmic and mitochondrial iron–sulfur (Fe—S) cluster‐mediated aconitase activity and abnormal expression of iron homeostasis–related proteins. Mechanistically, mass spectrometry analysis identified DOX‐treatment induces p53‐dependent degradation of Parkinsonism associated deglycase (Park7) which results in iron homeostasis dysregulation. Park7 counteracts iron overload by regulating iron regulatory protein family transcription while blocking mitochondrial iron uptake. Knockout of p53 or overexpression of Park7 in cardiomyocytes remarkably restores the activity of Fe—S cluster and iron homeostasis, inhibits ferroptosis, and rescues cardiac function in DOX treated animals. These results demonstrate that the iron homeostasis plays a key role in DoIC ferroptosis. Targeting of the newly identified p53–Park7 signaling axis may provide a new approach to prevent DoIC.

## Graphical abstract

Ferroptosis is demonstrated to play a role in doxorubicin (DOX)‐induced cardiotoxicity (DoIC). This study shows that upon DOX challenge, myocardial Park7, which binds to Fe—S clusters and acts upstream of the iron homeostasis pathway, is degraded in a p53‐dependent manner and results in iron homeostatic dysregulation. The novel pathway proposes a new perspective on the pathogenesis of DoIC.

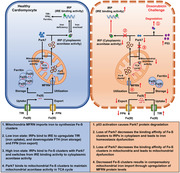

## 1 Introduction

The advancements in early detection and treatment have resulted in an increase in survival of cancer patients.^[^ ^1^ ^]^ However, adverse reactions due to chemotherapy are common, where cardiotoxicity can be life‐threatening.^[^ ^2^ ^]^ Cardiovascular mortality in cancer patients accounts for 11.3% of death.^[^ ^3^ ^]^ Anthracyclines, represented by doxorubicin (DOX), are a class of cytotoxic agents widely used in the treatment of various solid tumor and hematological malignancies, that has been shown to exhibit cardiotoxicity.^[^ ^4^ ^]^ The incidence of DOX‐related heart failure has been shown to be dose dependent: 5% at a cumulative dose of 400 mg m^−2^ and 26% at a dose of 550 mg m^−2^.^[^ ^5^ ^]^ However, a 13 years longitudinal study shows that subclinical events occurred in about 30% of the patients when treated with a low DOX doses of 180–240 mg m^−2^,^[^ ^6^ ^]^ suggesting better understanding of DOX‐induced cardiotoxicity (DoIC) is needed.

Ferroptosis is a novel iron‐dependent form of regulated cell death characterized by oxidative damage to membrane‐containing components.^[^ ^7^ ^]^ Intracellular iron accumulation promotes reactive oxygen species (ROS) production and activates lipoxygenases which attack membranes. Ferroptosis can be blocked by iron chelators or lipid peroxidation scavengers. Recently, ferroptosis has been demonstrated to play a role in DoIC.^[^ ^8^ ^]^ Although early study has reported that excess iron was accumulated in cardiomyocytes during DoIC,^[^ ^9^ ^]^ the underlying mechanism remains unknown.

In mammalian cells, iron is imported mainly through the high‐affinity transferrin receptor (TfR) and excreted through ferroportin (FPN). Excess iron is stored within the nanocavity of ferritin heteropolymers consists of heavy (FTH) and light (FTL) chains.^[^ ^10^ ^]^ Cellular iron homeostasis is orchestrated post‐transcriptionally by iron regulatory protein family (IRPs).^[^ ^10^ ^]^ By binding to iron response element (IRE), IRPs upregulate the expression of TfR and downregulates the expression of ferritin and FPN to maintain intracellular iron levels. The binding of IRPs to IRE is dependent on the level of iron–sulfur (Fe—S) clusters, which are synthesized in mitochondria through Fe—S cluster assembly machinery using ferrous ions that are imported into the mitochondria by mitoferrin (MFRN).^[^ ^11^ ^]^ Upon iron overload, intracellular Fe—S increases. Upon Fe—S binding, IRP1 loses its IRE‐binding activity and is converted into cytosolic aconitase state,^[^ ^12^ ^]^ whereas, IRP2 is ubiquitinated and degraded by SKP1‐CUL1‐FBXL5 ubiquitin ligase protein complex^[^ ^13^ ^]^ where FBXL5 activity is Fe—S sensitive.^[^ ^14^ ^]^ These events lower cellular iron uptake and increases iron storage and export. Inversely, decrease in Fe—S cluster levels can activate compensatory iron uptake and result in mitochondrial iron overload.^[^ ^15^ ^]^ Although the dysregulation of Fe—S levels may drive IRPs‐IRE dependent ferroptosis,^[^ ^16^, ^17^ ^]^ how this pathway is regulated in DoIC remains to be elucidated.

Parkinsonism associated deglycase (Park7) is a widely expressed protein originally identified in the dopaminergic neurons with the protective effect against Parkinson's disease.^[^ ^18^ ^]^ Similar to non‐dividing neurons, it has been shown that cardiac Park7 is dramatically down‐regulated in end‐stage human heart failure samples.^[^ ^19^ ^]^ In addition, Park7 has been suggested to suppress ferroptosis by regulating S‐adenosyl homocysteine hydrolase that confer protection to cancer cells against ferroptosis.^[^ ^20^ ^]^ Park7 can be post‐transcriptionally regulated by p53,^[^ ^21^ ^]^ which is significantly activated by DOX in cardiomyocytes.^[^ ^22^ ^]^ p53‐induced upregulation in death receptors potentially plays a role in increased cardiomyocyte apoptosis in DoIC.^[^ ^22^ ^]^ Besides, recent studies show that p53 is instrumental in regulating iron homeostasis in cancer cells.^[^ ^23^ ^]^ Whether the p53–Park7 axis plays a role in DOX‐induced iron homeostasis dysfunction in cardiomyocytes remains to be elucidated.

Here, using DoIC mouse model, we demonstrate that ferroptosis plays a pivotal role in DoIC both in vivo and in vitro. In healthy cardiomyocytes, we demonstrate that secondary iron overload, induced by ferric ammonium citrate (FAC) treatment,^[^ ^24^ ^]^ triggers series of Fe—S dependent pathway to lower iron uptake while increase iron removal and storage; however, DOX‐challenge results in iron homeostatic dysregulation. Using mass spectrometry, we identify and show Park7, which prevents iron overload by maintaining the activity of cytoplasmic and mitochondrial Fe—S clusters, is significantly degraded in DOX treated cardiomyocytes. Molecularly, Park7 protein is bound by p53 and its ubiquitination/degradation is induced. Last, we show that restoration of Park7 or knockdown of p53 in cardiomyocytes is capable of preventing DoIC‐induced ferroptosis. Together, we identify the importance of p53–Park7 regulation in iron homeostasis and its implications for preventing DoIC‐induced ferroptosis.

## 2 Results

### 2.1 Blocking Myocardial Ferroptosis Significantly Alleviates DoIC

**Figure 1:**
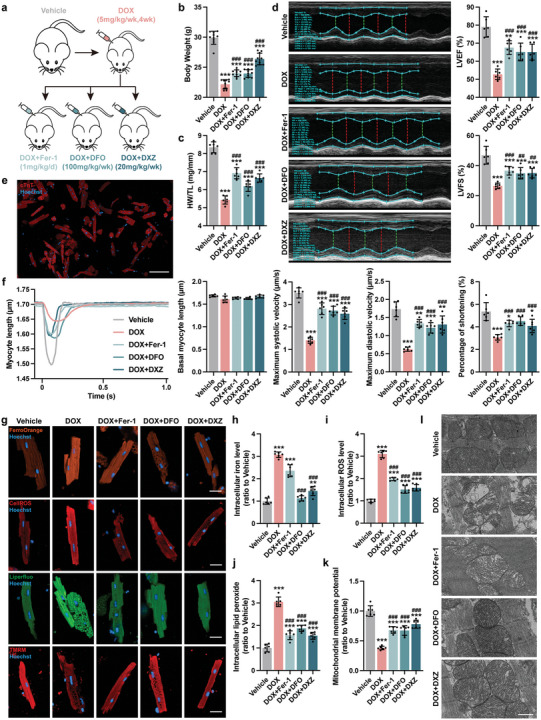
Blocking myocardial ferroptosis significantly alleviates DoIC in vivo. a) Experiment design. A chronic DoIC mouse model was established in C57BL/6J mice, and Fer‐1, DFO, and DXZ were used for anti‐ferroptosis treatment during the DoIC model. All tests and analysis were implemented 2 weeks after the last DOX injection. b) Body weight of mice (*n* = 10). c) Ratio of heart weight to tibia length (HW/TL; *n* = 6). d) Representative M‐mode images of transthoracic echocardiography, and quantification of left ventricular ejection fraction (LVEF) and left ventricular fraction shortening (LVFS; *n* = 6). e) Representative micrographs of isolated AMCMs stained with cTnT (scale bars, 100 µm). f) Representative tracings of sarcomere length of isolated AMCMs using an Ionoptix HTS system, and calculation of basal myocyte length, maximum systolic velocity, maximum diastolic velocity, and percentage of shortening of AMCMs (*n* = 6). g) Representative micrographs of intracellular iron level (FerroOrange staining), ROS (CellROS staining), lipid peroxide (Liperfluo staining), and mitochondrial membrane potential (TMRM staining) in AMCMs (scale bars, 20 µm), and the quantitative analysis of the fluorescence intensities were shown in (h–k) (*n* = 6). l) Representative micrographs of heart tissues examined by transmission electron micrographs (scale bars, 500 nm). The data are expressed as mean ± SD and analyzed using one‐way ANOVA followed by Tukey's post hoc test, *p < 0.05, **p < 0.01, and ***p < 0.001 versus Vehicle group; #*p* < 0.05, ##*p* < 0.01, and ###*p* < 0.001 versus DOX group.

To study the role of ferroptosis in DoIC, we used the well‐established DoIC mouse model and co‐treated DOX animals with ferrostatin‐1 (Fer‐1; a lipid peroxide scavenger), deferoxamine (DFO; an iron chelator), or dexrazoxane (DXZ; the only FDA‐approved drug for DoIC treatment partially via iron chelating) (**Figure** **1**a). Compared to vehicle group, DOX animals exhibited decreased body weight (Figure 1b and Figure S1a, Supporting Information) and heart weight (Figure 1c). Functionally, DOX animals exhibited poor systolic function marked by reduced left ventricular ejection fraction (LVEF), left ventricular fraction shortening (LVFS), and left ventricular global longitudinal strain (GLS) (Figure 1d and Figure S1b, Supporting Information). Histologically, DOX treatment significantly damaged myocardial tissue evidenced by disorganized myofibrillar arrangements and increased fibrosis (Figure S1c,d, Supporting Information). In single adult mouse cardiomyocytes (AMCMs) isolated using Langendorff perfusion method (Figure 1e), DOX treatment resulted in a significant decrease in systolic and diastolic function compared to vehicle group (Figure 1f and Figure S1e, Supporting Information). All these phenotypes were partially reversed by Fer‐1, DFO, and DXZ treatments (Figure 1b–f and Figure S1a–e, Supporting Information).

Next, we examined iron levels and downstream markers of ferroptosis in DOX treated cardiomyocytes. Compared to vehicles, DOX heart tissues exhibited an increase in Fe^2+^ and Fe^3+^ levels (Figure S1f, Supporting Information), as well as an increase in plasma and cardiac malondialdehyde (MDA) levels (Figure S1g, Supporting Information).^[^ ^25^ ^]^ In isolated DOX AMCMs, an increase in iron level (Figure 1g,h), intracellular reaction oxygen species (ROS) (Figure 1g,i), and lipid peroxidate levels (Figure 1g,j) were observed compared to vehicle AMCMs. Molecularly, the classical ferroptotic marker Ptsg2 was significantly induced in DOX AMCMs compared to vehicle AMCMs (Figure S1h, Supporting Information). DOX challenged AMCMs exhibited a decrease in mitochondrial membrane potential (Figure 1g,k) and mitochondrial ultrastructure alterations including ridge breakage and vacuolization (Figure 1i) suggestive of mitochondrial dysfunction. Fer‐1 treatment, which acts through lipid peroxide scavenging, partially alleviated the abovementioned ferroptotic phenotypes except iron accumulation; DFO and DXZ treatments, which eliminates intracellular iron, prevented activation of the ferroptosis pathway in DOX treated cardiomyocytes (Figure 1g–i and Figure S1f–h, Supporting Information).

**Figure 2:**
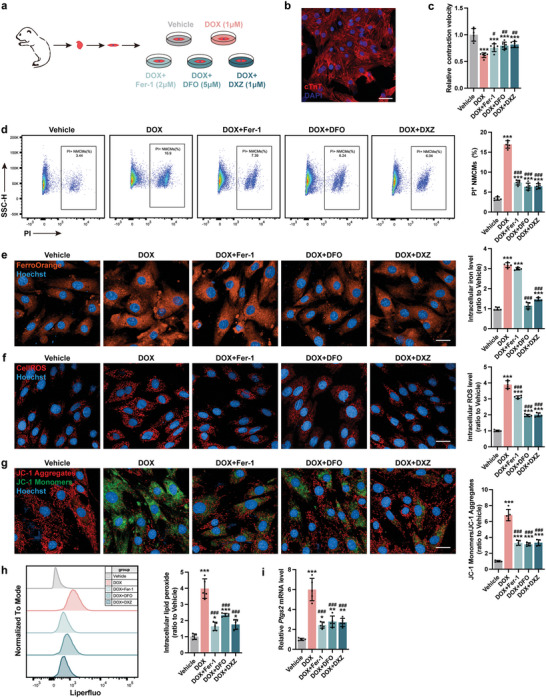
Blocking myocardial ferroptosis significantly alleviates DoIC in vitro. a) Experiment design for DoIC in neonatal murine cardiomyocytes (NMCMs) and Fer‐1, DFO, and DXZ were used for anti‐ferroptosis treatment prior to DOX challenge. b) Representative micrographs of isolated neonatal murine cardiomyocytes (NMCMs) stained with cTnT (scale bars, 50 µm). c) Contractile velocity of NMCMs extrapolated from live cell imaging (*n* = 6). d) The percentage of PI^+^ NMCMs were calculated using flow cytometry (*n* = 5). e) Representative micrographs and quantification of intracellular iron level (FerroOrange staining) in NMCMs (scale bars, 20 µm; *n* = 5). f) Representative micrographs and quantification of intracellular ROS (CellROS staining) in NMCMs (scale bars, 20 µm; *n* = 5). g) Representative micrographs and quantification of mitochondrial membrane potential (JC‐1 staining) in NMCMs (scale bars, 20 µm; *n* = 5). h) Quantification of intracellular lipid peroxide in NMCMs by flow cytometry (*n* = 5). i) Ptgs2 mRNA expression in NMCMs (*n* = 5). The data are expressed as mean ± SD and analyzed using one‐way ANOVA followed by Tukey's post hoc test, *p < 0.05, **p < 0.01, and ***p < 0.001 versus Vehicle group; #*p* < 0.05, ##*p* < 0.01, and ###*p* < 0.001 versus DOX group.

To interrogate the mechanistic underpinnings of iron accumulation and mitochondrial dysfunction, we isolated and used neonatal mouse cardiomyocytes (NMCMs) for our subsequent studies (**Figure** **2**a,b). Consistently, DOX treatment resulted in a decline in contractile speed and cell viability in NMCMs and was rescued by blocking ferroptosis (Figure 2c,d). DFO and DXZ treatments, but not Fer‐1 treatment, reduced DOX‐induced iron accumulation in NMCMs (Figure 2e). In keep with our mouse data, all three interventions inhibited DOX‐induced ROS (Figure 2f) and lipid peroxide production (Figure 2h), restored mitochondrial membrane potential (Figure 2g), as well as reversed Ptgs2 expression (Figure 2i). Together, these results show DOX‐induced iron overload drives mitochondrial dysfunction and myocardial dysfunction in cardiomyocytes.

### 2.2 DOX Impairs Iron Homeostasis in Cardiomyocytes

**Figure 3:**
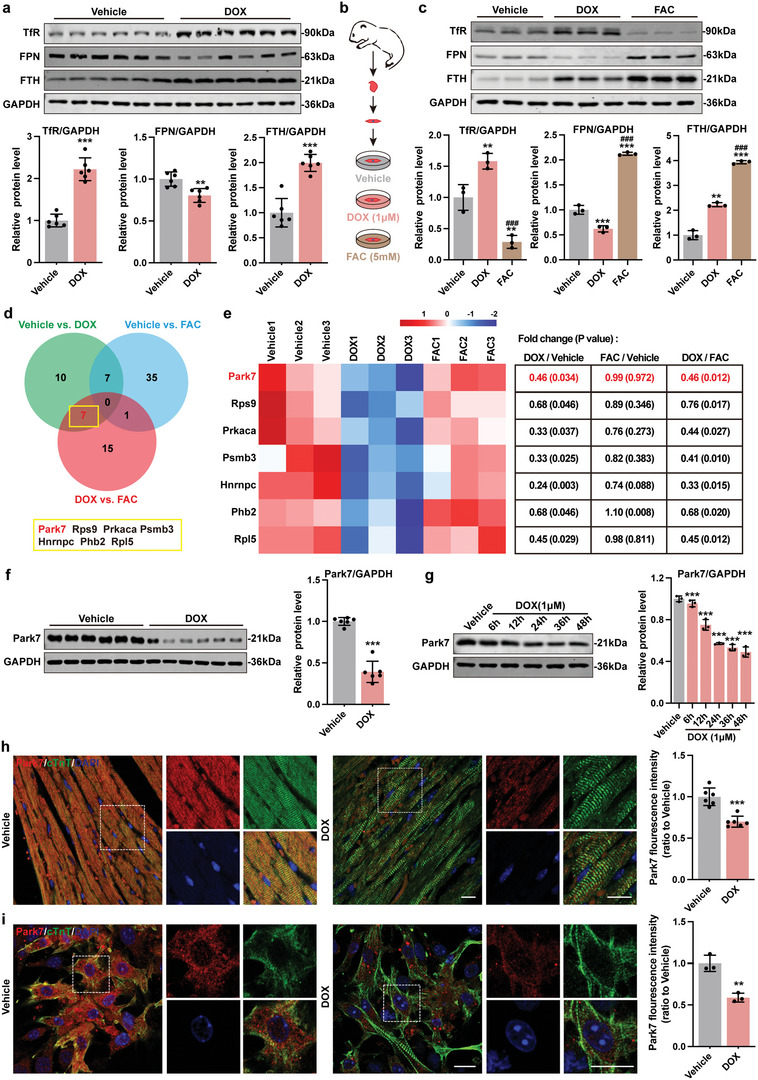
DOX impairs iron homeostasis in cardiomyocytes and can be associated with downregulation of Park7. a) Representative immunoblots and quantitative analysis of iron homeostasis–related proteins in AMCMs from chronic mice DoIC model (*n* = 6). b) Experiment design for DoIC and FAC based iron overload model in NMCMs. c) Representative immunoblots and quantitative analysis of iron homeostasis–related proteins in NMCMs (*n* = 3). d) Venn analysis of differentially expressed proteins in NMCMs (Proteins of a 1.2‐fold change (≥1.2 or ≤0.83) of Coverage with *P* < 0.05 cutoff). e) Heatmap and the specific fold change and *P*‐value of DOX‐dysregulated proteins. f) Representative blots and quantitative analysis of Park7 protein level in AMCMs from chronic mice DoIC model (*n* = 6). g) Representative immunoblots and quantitative analysis of Park7 protein level in NMCMs treated with 1 µm DOX in different times (*n* = 3). h) Representative micrographs and quantitative analysis of Park7 protein level in heart tissues from chronic mice DoIC model detected using immunofluorescent staining (scale bars, 20 µm; *n* = 6). i) Representative micrographs and quantitative analysis of Park7 protein level in NMCMs after DOX (1 µm, 24 h) treated detected using immunofluorescent staining (scale bars, 30 µm; *n* = 3). The data are expressed as mean ± SD and analyzed using Student's *t*‐test or one‐way ANOVA followed by Tukey's post hoc test. *p < 0.05, **p < 0.01, and ***p < 0.001 versus Vehicle group; #*p* < 0.05, ##*p* < 0.01, and ###*p* < 0.001 versus DOX group.

Recent advances demonstrated the importance of iron homeostasis regulated by TfR (iron absorption), FTH (iron storage), and FPN (iron excretion) in preventing ferroptosis.^[^ ^26^ ^]^ Compared to vehicle group, AMCMs from DOX animals exhibited an increase in TfR and FTH, and a decrease in FPN protein expression (**Figure** **3**a). To determine if iron overload alone can recapitulate DOX‐induced damage, we treated NMCMs with FAC to induce secondary iron overload (Figure 3b).^[^ ^24^ ^]^ Although FAC treatment was capable of inducing intracellular iron accumulation similar to DOX (Figure S2a, Supporting Information), the changes in iron homeostasis–related protein expressions were quite different. Compared to the vehicle group, FAC treatment resulted in a decrease in TfR, and an increase in FPN and FTH proteins, opposite of DOX treated NMCMs (Figure 3c). These findings suggest that FAC‐induced iron overload activates myocardial iron clearance pathway yet this cardioprotection is lost in DOX treated cardiomyocytes.

### 2.3 DOX‐Induced Downregulation of Park7 Results in Loss of Iron Clearance Protection in Cardiomyocytes

Given that iron overload in DOX treated cardiomyocytes likely occurs in conjunction to mitochondrial dysfunction, we hypothesized that DOX treatment must be capable of silencing cardioprotective iron regulation that is seen in our FAC‐treated cardiomyocytes. To identify the molecular switch that inhibits iron clearance upon DOX challenge, we performed mass spectrometry analyses in NMCMs treated with DOX, FAC, and vehicle control. Using a 1.2 protein coverage fold change cutoff regardless of *P*‐value, we identified 96 potential DOX‐dysregulated proteins in NMCMs (Figure S2b,d, Supporting Information) that show an enriched in metabolism and ion transport‐related functions (Figure S2c, Supporting Information). When we implemented a *P* < 0.05 cutoff, only seven proteins remain differentially regulated (Figure 3d,e). Among them, Hnrnpc and Phb2 have been reported to promote ferroptosis thus downregulation in DOX treatment did not fit with our hypothesis.^[^ ^27^, ^28^ ^]^ No evidence of ferroptosis nor iron homeostasis were found for Rps9, Rpl5, Prkaca, and Psmb3. Park7 was chosen for further characterization for its ability to stabilize and increase intracellular Fe—S cluster levels in prokaryotic systems. When intracellular iron is high, Fe—S cluster level rises and cells counteract by decreasing iron import (downregulate TfR) while increasing iron clearance and storage (upregulate FTH and FPN). To accomplish this, Fe—S clusters bind to IRP1 that converts its IRE‐binding transcriptional activity into cytosolic aconitase state and induce SKP1‐CUL1‐FBXL5^[^ ^13^, ^14^ ^]^ dependent IRP2 degradation.^[^ ^10^ ^]^ Loss of IRP1 and IRP2 transcriptional activity results in downregulation of TfR (iron import) and upregulation of FTH and FPN (iron storage and removal).

To confirm if DOX treatment downregulates Park7 protein levels, we first examined Park7 expression in AMCMs (Figure 3f) and NMCMs (Figure 3g). DOX challenge caused a decrease in Park7 proteins (Figure 3f,g) without altering Park7 mRNA levels (Figure S2e,f, Supporting Information) suggestive of post‐translational regulation. Immunofluorescence staining in heart sections and NMCMs confirms this observation (Figure 3h,i). Importantly, compared to DOX‐challenged NMCMs, iron overload alone (FAC condition) did not alter Park7 protein levels (Figure S2g, Supporting Information). These results suggest that DOX treatment results in Park7 protein downregulation at the post‐translational level that is independent of iron overload.

**Figure 4:**
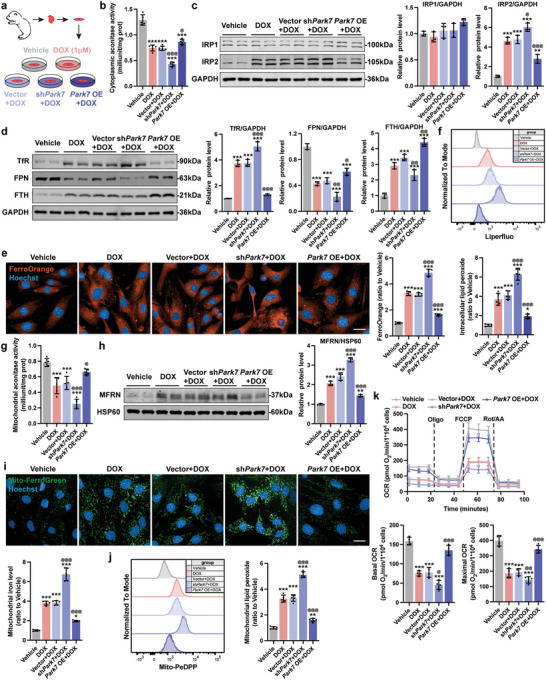
DOX‐induced downregulation of Park7 results in loss of iron clearance and promotes mitochondrial iron overload in cardiomyocytes. a) Experiment design for downregulation or overexpression of Park7 in NMCMs prior to DOX (1 µm, 24 h) challenge. b) The cellular aconitase activity in NMCMs (*n* = 5). c) Representative immunoblots and quantitative analysis of IRP1 and IRP2 protein level in NMCMs (*n* = 3). d) Representative immunoblots and quantitative analysis of iron homeostasis–related proteins in NMCMs (*n* = 3). e) Representative micrographs and quantification of intracellular iron level (FerroOrange staining) in NMCMs (scale bars, 20 µm; *n* = 5). f) Quantification of intracellular lipid peroxide in NMCMs by flow cytometry (*n* = 5). g) The mitochondrial aconitase activity in NMCMs (*n* = 5). h) Representative immunoblots and quantitative analysis of MFRN protein level in mitochondria of NMCMs (*n* = 3). i) Representative micrographs and quantification of mitochondrial iron level (Mito‐FerroGreen staining) in NMCMs (scale bars, 20 µm; *n* = 5). j) Quantification of mitochondrial lipid peroxide in NMCMs by flow cytometry (*n* = 5). k) Real‐time oxygen consumption rates (OCR) and calculated basal and maximal respiration rates in NMCMs (*n* = 4). The data are expressed as mean ± SD and analyzed using one‐way ANOVA followed by Tukey's post hoc test, *p < 0.05, **p < 0.01, and ***p < 0.001 versus Vehicle group; @*p* < 0.05, @@*p* < 0.01, and @@@*p* < 0.001 versus Vector+DOX group.

To explore whether DOX‐induced Park7 downregulation is the driver of iron overload and ferroptosis in cardiomyocytes, we overexpressed (Park7 OE) and knockdown (shPark7) Park7 levels using lentiviruses prior to DOX challenge (**Figure** **4**a). The infection efficiencies were assayed by RT‐PCR and immunoblotting (Figure S3a,b, Supporting Information). Functionally, shPark7 treatment of DOX NMCMs further dampened myocardial contractility (Figure S3c, Supporting Information) and decreased cell viability (Figure S3d, Supporting Information) while Park7 overexpression did the reverse compared to DOX treatment. Using cytoplasmic aconitase activity as a readout of Fe—S cluster‐IRPs binding affinity, DOX challenge significantly reduced cytoplasmic aconitase activity compared to vehicle (Figure 4b). Further, knockdown or overexpression of Park7 prior to DOX treatment resulted in decrease or increase of cytoplasmic aconitase activity, respectively (Figure 4b). Unlike IRP1, IRP2 protein levels significantly increased in the DOX group, and were further aggravated by shPark7 but reversed by Park7 overexpression (Figure 4c). Next, we examined if disruption of Park7 expression can affect the expression of IRP‐IRE–regulated iron homeostasis–related proteins. DOX treatment in shPark7 NMCMs resulted in further upregulation of TfR and downregulation of FPN and FTH (Figure 4d), further accumulation of intracellular iron (Figure 4e), further increase in ROS (Figure S3e, Supporting Information) and lipid peroxides (Figure 4f), as well as the upregulation of Ptgs2 (Figure S3f, Supporting Information). In contrast, these phenotypes were partially reversed in DOX treated Park7 OE NMCMs (Figure 4d–f and Figure S3e,f, Supporting Information). Together, these results provide evidence that DOX challenge results in Park7 protein levels which result in iron dysregulation.

### 2.4 DOX‐Induced Downregulation of Park7 Promotes Mitochondrial Iron Overload and Mitochondrial Dysfunction

Given that intracellular ferrous ions are first imported into mitochondria by MFRN for Fe—S synthesis,^[^ ^11^ ^]^ and cytosolic and mitochondrial Fe—S cluster levels help the cells sense and maintain iron homeostasis, we examined the effect of DOX challenge on mitochondrial respiration and mitochondrial aconitase activity. In DOX treated shPark7 NMCMs, mitochondrial aconitase activity (surrogate measure of the binding affinity of mitochondrial Fe—S cluster) further decreased while Park7 OE rescued (Figure 4g). Downregulated mitochondrial Fe—S cluster activity promoted compensatory iron uptake by mitochondria evident by increased MFRN protein expression and mitochondrial iron accumulation (Figure 4h,i). In addition, we found that shPark7 further exacerbated DOX‐induced increase in mitochondrial superoxide (Figure S3g, Supporting Information) and mitochondrial lipid peroxide (Figure 4j), and decrease in mitochondrial membrane potential (Figure S3h, Supporting Information) in NMCMs; whereas, Park7 OE conferred protection against DOX challenge (Figure 4h–j and Figure S3g,h, Supporting Information). To assess mitochondrial function, we performed mitochondrial respiration stress test using a Seahorse XFe96 bioanalyzer. In agreement to prior observations,^[^ ^29^ ^]^ we observed a significant reduction of basal and maximal respiration upon DOX challenge (Figure 4k). This reduction was further exacerbated in DOX treated shPark7 NMCMs but reversed in Park7 OE NMCMs (Figure 4k). Together, these findings suggest that downregulation of Park7 by DOX challenge results in a compensatory induction of mitochondrial Fe—S clusters formation via MFRN, leading to mitochondrial iron overload and dysfunction.

### 2.5 p53 Is Responsible for DOX‐Induced Downregulation of Park7

**Figure 5:**
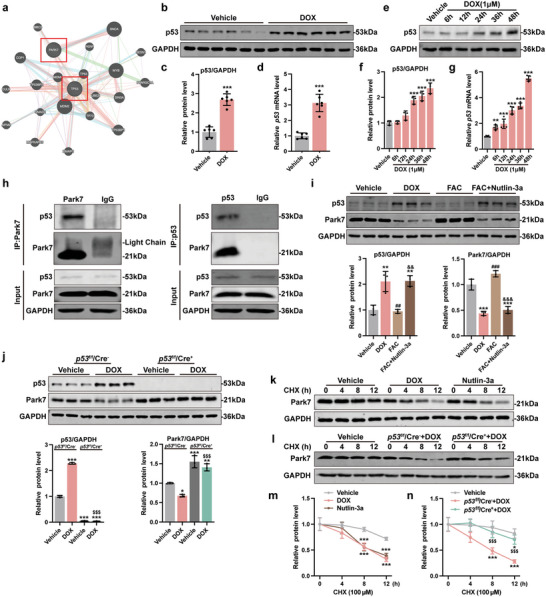
p53 is responsible for DOX‐induced downregulation of Park7. a) Protein interaction prediction of Park7 and p53 in GeneMANIA. b,c) Representative immunoblots and quantitative analysis of p53 protein level in AMCMs from chronic mice DoIC model (*n* = 6). d) p53 mRNA expression level in AMCMs from chronic mice DoIC model (*n* = 6). e,f) Representative immunoblots and quantitative analysis of p53 protein level in NMCMs treated with 1 µm DOX in different times (*n* = 3). g) p53 mRNA expression level in NMCMs treated with 1 µm DOX in different times (*n* = 5). h) Coimmunoprecipitation was performed to determine the interaction between endogenous Park7 and p53 in NMCMs. i) Representative immunoblots and quantitative analysis of Park7 and p53 protein level in NMCMs treated with DOX, FAC, or FAC plus Nutlin‐3a (*n* = 3). j) Representative immunoblots and quantitative analysis of Park7 and p53 protein level in NMCMs isolated from p53^f/f^/Cre^−^ and p53^f/f^/Cre^+^ mice (*n* = 3). k) Representative immunoblots of Park7 protein level in NMCMs treated with DOX or Nutlin‐3a followed by a time‐dependent CHX treatment. The quantitative analysis was shown in (m) (*n* = 3). l) Representative immunoblots of Park7 protein level in NMCMs isolated from p53^f/f^/Cre^−^ and p53^f/f^/Cre^+^ mice treated with DOX followed by a time‐dependent CHX treatment. The quantitative analysis was shown in (n) (*n* = 3). The data are expressed as mean ± SD and analyzed using Student's *t*‐test or one‐way ANOVA followed by Tukey's post hoc test, *p < 0.05, **p < 0.01, and ***p < 0.001 versus Vehicle group; #*p* < 0.05, ##*p* < 0.01, and ###*p* < 0.001 versus DOX group; &&&*p* < 0.001 versus FAC group; $$$*p* < 0.001 versus p53^f/f^/Cre^−^+DOX group.

Next, we asked how DOX challenge drives the decrease of Park7 protein levels in cardiomyocytes. To accomplish this, we used GeneMANIA (http://genemania.org/) to identify potential interacting proteins of Park7 (**Figure** **5**a). Interestingly, p53 came up as a potential binding partner and it has been shown in mouse embryonic fibroblasts that p53 can negatively regulate Park7 protein levels,^[^ ^21^ ^]^ but whether p53 can cause Park7 protein degradation remains unknown. We confirmed that a decrease in Park7 protein expression (Figure 3e–h) was accompanied with an increase in p53 protein levels in DOX treated AMCMs and NMCMs (Figure 5b–g). Given that DOX‐induced Park7 downregulation occurs post‐translationally, we hypothesized that p53 binds to Park7 to induce its degradation. To verify if p53 interacts with Park7, we performed co‐immunoprecipitation (Co‐IP) in NMCMs. Indeed, endogenous p53–Park7 interaction can be observed using either p53 or Park7 as bait (Figure 5h). To demonstrate causality, we induced p53 with Nutlin‐3a (a MDM2 inhibitor) in FAC treated NMCMs. Surprisingly, compared to the vehicle and FAC group which is p53 low and Park7 high, the induction of p53 protein in iron overloaded NMCMs was sufficient to reduce Park7 levels (FAC+Nutlin‐3a group) similar to DOX‐treated NMCMs (Figure 5i). To assess causality, we generated myocardial specific p53 knockout animals (p53^CKO^) by crossing p53^flox/flox^ animals with Myh6‐Cre animals as previously described.^[^ ^30^ ^]^ In p53^CKO^ NMCMs, we observed an increase in basal Park7 protein levels and DOX‐treatment did not result in Park7 protein reduction (Figure 5j). To determine how p53 regulates the stability of Park7 proteins, we treated NMCMs with protein synthesis inhibitor cycloheximide (CHX) and examined Park7 protein levels in a time course. Compared to the vehicle control, DOX and Nutlin‐3a treatment accelerated degradation of Park7 protein levels (Figure 5k,m). This acceleration of Park7 protein degradation returned to basal levels in DOX treated p53^CKO^ NMCMs (Figure 5l,n). Next, we hypothesize that p53 binding of Park7 induces Park7 degradation through ubiquitination. Upon p53 activation (DOX or Nutlin‐3a), we observed a decrease in Park7 protein levels and an accumulation of ubiquitinated proteins in NMCMs when we blocked proteasome with MG132 (Figure S4a, Supporting Information). Compared to control NMCMs, DOX treatment in p53^CKO^ was incapable of inducing Park7 protein downregulation and accumulation of ubiquitinated proteins (Figure S4b, Supporting Information). Collectively, our data suggest that p53 downregulates Park7 protein levels through ubiquitination which silences iron clearance pathway.

To elucidate whether p53‐dependent Park7 degradation precedes or is dependent of iron overload, we treated NMCMs with Nutlin‐3a just to induce p53‐dependent Park7 degradation and examined if Park7 OE is sufficient to rescue (Figure S5a,b Supporting Information). Park7 OE rescued cytoplasmic and mitochondrial aconitase activity (Figure S5c, Supporting Information) and reversed IRP2, TfR, FPN, FTH, and MFRN protein levels induced by Nutlin‐3a challenge (Figure S5d,e, Supporting Information). Interestingly, the intracellular and mitochondrial iron levels were slightly higher in Nutlin‐3a NMCMs, which were reversed by Park7 OE (Figure S5f,g, Supporting Information). These results suggest that degradation of Park7 by p53 occurs prior to iron dysregulation and iron accumulation.

### 2.6 p53 Activation Recapitulates Ferroptosis Phenotype in FAC‐Treated NMCMs

**Figure 6:**
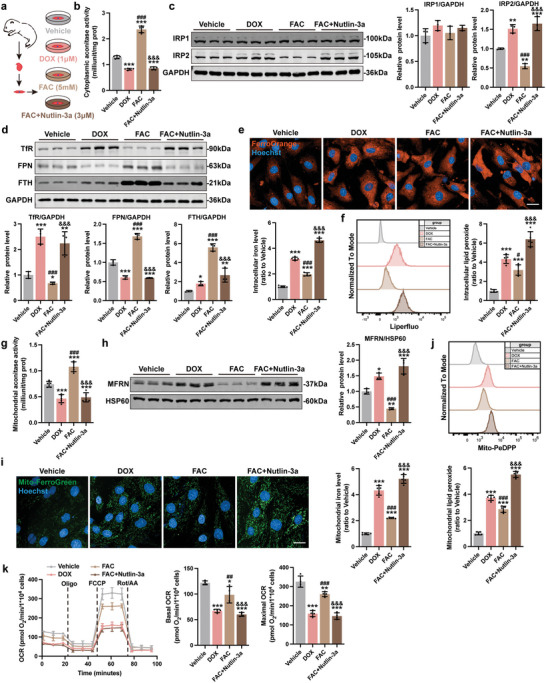
p53 activation recapitulates ferroptosis phenotype in FAC‐treated NMCMs. a) Experiment design for p53 activation using Nutlin‐3a in FAC treated NMCMs. b) The cellular aconitase activity in NMCMs (*n* = 5). c) Representative immunoblots and quantitative analysis of IRP1 and IRP2 protein level in NMCMs (*n* = 3). d) Representative immunoblots and quantitative analysis of iron homeostasis–related proteins in NMCMs (*n* = 3). e) Representative micrographs and quantification of intracellular iron level (FerroOrange staining) in NMCMs (scale bars, 20 µm; *n* = 5). f) Quantification of intracellular lipid peroxide in NMCMs by flow cytometry (*n* = 5). g) The mitochondrial aconitase activity in NMCMs (*n* = 5). h) Representative immunoblots and quantitative analysis of MFRN protein level in mitochondria of NMCMs (*n* = 3). i) Representative micrographs and quantification of mitochondrial iron level (Mito‐FerroGreen staining) in NMCMs (scale bars, 20 µm; *n* = 5). j) Quantification of mitochondrial lipid peroxide in NMCMs by flow cytometry (*n* = 5). k) Real‐time oxygen consumption rates (OCR) and calculated basal and maximal respiration rates in NMCMs (*n* = 4). The data are expressed as mean ± SD and analyzed using one‐way ANOVA followed by Tukey's post hoc test, *p < 0.05, **p < 0.01, and ***p < 0.001 versus Vehicle group; #*p* < 0.05, ##*p* < 0.01, and ###*p* < 0.001 versus DOX group; &&&*p* < 0.001 versus FAC group.

To further verify p53–Park7 signaling axis drives ferroptosis in DoIC, we treated FAC NMCMs with Nutlin‐3a (**Figure** **6**a). Functionally, a decrease in myocardial contractility (Figure S6a, Supporting Information) and cell viability (Figure S6b, Supporting Information) were observed in FAC+Nutlin‐3a NMCMs compared to FAC controls. In p53‐activated FAC NMCMs, we were able to recapitulate iron dysregulation evidenced by reduced cytoplasmic aconitase activity (Figure 6b), upregulation of IRP2 (Figure 6c) and TfR (Figure 6d), downregulation of FPN and FTH (Figure 6d), and aggravated intracellular iron accumulation (Figure 6e) compared to FAC NMCMs. Further, an increase in cellular ROS (Figure S6c, Supporting Information) and lipid peroxide (Figure 6f) levels, and upregulation of Ptgs2 (Figure S6d, Supporting Information) were observed in FAC+Nutlin‐3a NMCMs compared to FAC controls. Likewise, compared to the FAC controls, FAC+Nutlin‐3a resulted in a significant decrease in mitochondrial aconitase activity (Figure 6g), an increase in MFRN protein expression (Figure 6h), an increase in mitochondrial iron (Figure 6i), an increase in mitochondrial superoxide (Figure S6e, Supporting Information) and mitochondrial lipid peroxide (Figure 6j), a decrease in mitochondrial membrane potential (Figure S6f, Supporting Information), and a decrease in mitochondrial respiration capacity (Figure 6k). These data demonstrate that iron overload plus p53 activation, in absence of actual DNA damage caused by DOX, is sufficient in inducing Park7 downregulation, iron overload, and mitochondrial dysfunction.

**Figure 7:**
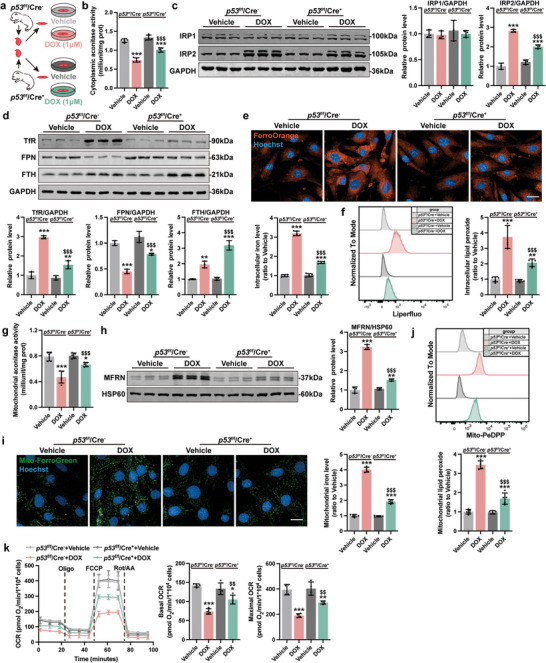
Knockout of p53 ameliorated DOX‐induced iron homeostasis dysfunction and ferroptosis in NMCMs. a) Experiment design for DOX treatment (1 µm, 24 h) in NMCMs isolated from p53^f/f^/Cre^−^ and p53^f/f^/Cre^+^ mice. b) The cellular aconitase activity in NMCMs (*n* = 5). c) Representative immunoblots and quantitative analysis of IRP1 and IRP2 protein level in NMCMs (*n* = 3). d) Representative immunoblots and quantitative analysis of iron homeostasis–related proteins in NMCMs (*n* = 3). e) Representative micrographs and quantification of intracellular iron level (FerroOrange staining) in NMCMs (scale bars, 20 µm; *n* = 5). f) Quantification of intracellular lipid peroxide in NMCMs by flow cytometry (*n* = 5). g) The mitochondrial aconitase activity in NMCMs (*n* = 5). h) Representative immunoblots and quantitative analysis of MFRN protein level in mitochondria of NMCMs (*n* = 3). i) Representative micrographs and quantification of mitochondrial iron level (Mito‐FerroGreen staining) in NMCMs (scale bars, 20 µm; *n* = 5). j) Quantification of mitochondrial lipid peroxide in NMCMs by flow cytometry (*n* = 5). k) Real‐time oxygen consumption rates (OCR) and calculated basal and maximal respiration rates in NMCMs (*n* = 4). The data are expressed as mean ± SD and analyzed using one‐way ANOVA followed by Tukey's post hoc test. *p < 0.05, **p < 0.01, and ***p < 0.001 versus Vehicle group; $*p* < 0.05, $$*p* < 0.01, and $$$*p* < 0.001 versus p53^f/f^/Cre^−^+DOX group.

Next, p53^CKO^ NMCMs were isolated and treated with DOX (**Figure** **7**a). p53 deficiency notably improved myocardial contractility (Figure S7a, Supporting Information) and cell viability (Figure S7b, Supporting Information) upon DOX challenge, and significantly prevented the onset of iron dysregulation: increase in cytoplasmic aconitase activity (Figure 7b), decrease in IRP2 (Figure 7c) and TfR protein levels (Figure 7d), increase in FTH and FPN protein levels (Figure 7d), and decrease in intracellular iron accumulation (Figure 7e). Further, reduced cellular ROS (Figure S7c, Supporting Information) and lipid peroxide levels (Figure 7f), and downregulation of Ptgs2 (Figure S7d, Supporting Information) were observed in p53^CKO^ NMCMs upon DOX challenge. Compared to WT NMCMs, DOX‐challenged p53^CKO^ NMCMs exhibited an increase in mitochondrial aconitase activity (Figure 7g), a decrease in MFRN protein level (Figure 7h), a decrease in mitochondrial iron accumulation (Figure 7i), a decrease in mitochondrial superoxide (Figure S7e, Supporting Information) and mitochondrial lipid peroxide (Figure 7j), an increase in mitochondrial membrane potential (Figure S7f, Supporting Information), and an increase in mitochondrial respiration capacity (Figure 7k). Together, these results show that p53 activation is necessary to drive ferroptosis in presence of iron overload.

### 2.7 Park7 Restoration Alleviates DOX Induced Myocardial Ferroptosis in Mice

**Figure 8:**
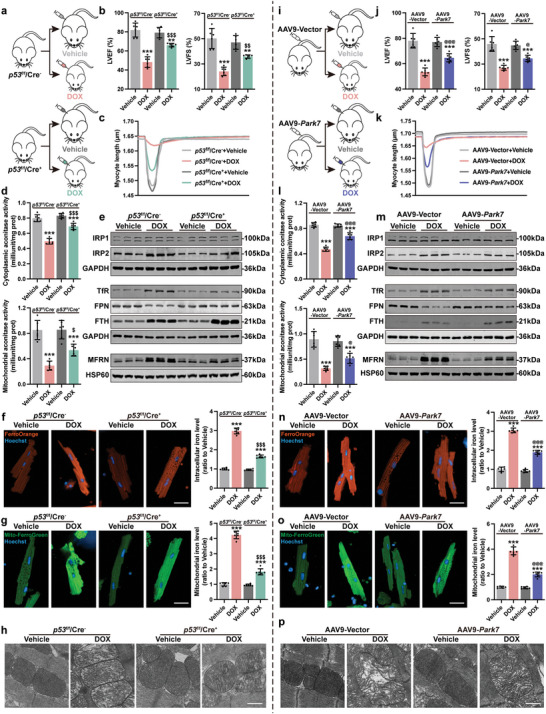
Park7 restoration alleviates DOX induced myocardial ferroptosis in mice. a,i) Experiment design for chronic DoIC mouse model established in p53 CKO and Park7 OE mice. b,j) Quantification of LVEF and LVFS measured from M‐mode transthoracic echocardiography (*n* = 6). c,k) Representative tracings of sarcomere length tracing of isolated AMCMs using an Ionoptix HTS system. d,l) The cellular and mitochondrial aconitase activity in AMCMs (*n* = 6). e,m) Representative immunoblots of cellular and mitochondrial iron homeostasis–related proteins in AMCMs. f,n) Representative micrographs and quantification of intracellular iron level (FerroOrange staining) in AMCMs (scale bars, 20 µm, *n* = 6). g,o) Representative micrographs and quantification of mitochondrial iron level (Mito‐FerroGreen staining) in AMCMs (scale bars, 20 µm, *n* = 6). h,p) Representative micrographs of heart tissues examined by transmission electron micrographs (scale bars, 500 nm). The data are expressed as mean ± SD and analyzed using one‐way ANOVA followed by Tukey's post hoc test, **p < 0.01 and ***p < 0.001 versus Vehicle group; $*p* < 0.05, $$*p* < 0.01, and $$$*p* < 0.001 versus p53^f/f^/Cre^−^+DOX group; @*p* < 0.05 and @@@*p* < 0.001 versus AAV9‐Vector+DOX group.

To test if restoration of Park7 protein levels will prevent the onset of DOX induced ferroptosis in vivo, we either challenged p53^CKO^ animals with DOX (**Figure** **8**a and Figure S8a, Supporting Information) or overexpressed Park7 in cardiomyocytes using an AAV9‐cTnT‐Park7 virus prior to DOX challenge (Figure 8i and Figure S9a, Supporting Information).

In keep with our NMCMs results, p53 deficiency significantly increased Park7 protein levels in AMCMs, which was not reduced upon DOX challenge (Figure S8a, Supporting Information). Both p53^CKO^ and Park7 OE animals exhibited delayed DOX‐induced body and heart weight loss (Figure S8b–d and S9b–d, Supporting Information). Functionally, p53 deficiency (Figure 8b and Figure S8e,f, Supporting Information) or Park7 OE (Figure 8j and Figure S9e,f, Supporting Information) prevented the onset of cardiac systolic dysfunction upon DOX challenge measured by LVEF, LVFS, and left ventricular GLS. Histologically, restoration of Park7 prevented DOX‐induced myofibrillar disorganization and cardiac fibrosis in p53^CKO^ (Figure S8g,h, Supporting Information) and Park7 OE hearts (Figure S9g,h, Supporting Information). Contractile functions were significantly better in DOX‐challenged p53^CKO^ (Figure 8c and Figure S8i, Supporting Information) and Park7 OE AMCMs (Figure 8k and Figure S9i, Supporting Information) compared to control AMCMs. Further, cytoplasmic and mitochondrial aconitase activities were partially alleviated DOX‐treated p53^CKO^ (Figure 8d) and DOX‐treated Park7 OE AMCMs (Figure 8l). Protein expressions of iron homeostasis–related proteins were also partially rescued in p53^CKO^ (Figure 8e and Figure S10a, Supporting Information) or Park7 OE AMCMs (Figure 8m and Figure S11a, Supporting Information) compared to controls. Further, p53 deficiency or Park7 OE prevented the accumulation of iron in heart tissues (Figures S10b and S11b, Supporting Information) and AMCMs (Figure 8f,n), rescued intracellular ROS (Figures S10c and S11c, Supporting Information) and lipid peroxidate levels (Figures S10d and S11d, Supporting Information), lowered plasma and cardiac MDA levels (Figures S10e and S11e, Supporting Information), and prevented Ptgs2 induction (Figures S10f and S11f, Supporting Information) observed in DOX‐treated control animals and AMCMs. Further, mitochondrial iron accumulation (Figure 8g,o), mitochondrial superoxide accumulation (Figures S10g and S11g, Supporting Information), and mitochondrial lipid peroxide accumulation (Figures S10h and S11h, Supporting Information) were all reversed in DOX‐treated p53^CKO^ and Park7 OE AMCMs compared to controls. We also observed healthier mitochondria in DOX‐treated p53^CKO^ and Park7 OE AMCMs evidenced by restoration of mitochondrial membrane potential (Figures S10i and S11i, Supporting Information) and improved mitochondrial ultrastructure (Figure 8h,p) compared to DOX‐treated controls. In summary, our results demonstrate that preventing p53‐dependent Park7 degradation can significantly prevent DOX induced iron overload and myocardial ferroptosis in cardiomyocytes.

## 3 Discussion

In this study, we demonstrate that p53–Park7 signaling axis plays a key role in myocardial iron homeostasis and its dysregulation is responsible for DoIC‐induced ferroptosis. In healthy cardiomyocytes, Park7 confers cardioprotection through the Fe—S cluster/IRP‐IRE regulatory pathway. Upon DOX challenge, p53 ubiquitinates Park7 leading to its degradation, and results in iron overload and myocardial ferroptosis.

It has been demonstrated that DOX challenge results in iron overload and leads to ferroptosis in cardiomyocytes,^[^ ^9^ ^]^ however, the severity and kinetics of DoIC is much greater than iron overload alone. Increase in dietary iron intake can further exacerbate DoIC in rats^[^ ^31^ ^]^ suggesting DoIC myocardial loss is mostly due to ferroptosis. Fang et al. first attributed the accumulation of intracellular iron to the degradation of heme via Nrf2 activation in acute DoIC model^[^ ^8^ ^]^ yet Nrf2 is a classic antioxidant factor that is cardioprotective during chronic DoIC.^[^ ^32^, ^33^ ^]^ Although ferritinophagy may contribute to iron overload,^[^ ^34^, ^35^ ^]^ our prior works demonstrating decreased autophagy flux in DOX treated cardiomyocytes^[^ ^36^ ^]^ suggest this is unlikely. Under high‐iron diet challenge, Fth^CKO^ animals exhibited aggravated ferroptosis levels^[^ ^37^ ^]^ highlighting the importance of iron homeostasis in preventing ferroptosis. Conversely, inhibition of TfR with neutralizing antibody reduced iron uptake and intracellular ROS formation in DOX‐treated endothelial cells^[^ ^38^ ^]^ while FPN‐dependent iron export has been shown to be anti‐ferroptosis.^[^ ^39^ ^]^ Together, these results suggest DoIC is likely due to dysregulation of iron homeostasis and ferroptosis.

Blocked iron homeostasis, regulated by the Fe—S cluster‐IRP‐IRE system, has been suggested in DOX challenged cardiomyocytes.^[^ ^40^ ^]^ Further, mitochondria is the major site of iron utilization for Fe—S clusters synthesis^[^ ^11^ ^]^ and recent work emphasized the role of mitochondrial iron overload in cardiomyocyte ferroptosis.^[^ ^8^, ^41^, ^42^ ^]^ In Friedreich's ataxia, lowered Fe—S cluster levels is perceived as mitochondrial iron deficiency and cells compensate by increasing mitochondrial iron uptake.^[^ ^15^ ^]^ Overexpression of ATP binding cassette subfamily B member 8, a mitochondrial iron export protein, partially reversed the progression of DoIC.^[^ ^42^ ^]^ Using mass spectrometry, here we identified Park7 as the key regulator in myocardial iron homeostasis. Park7 is expressed ubiquitously and was originally identified in dopaminergic neurons of familial Parkinson's disease patients.^[^ ^18^ ^]^ Chin et al. observed altered IRE gene transcript abundance in Park7 knockout zebrafish brain^[^ ^43^ ^]^ and Lin et al. reported a novel PARK7 homozygous mutation (c.390delA) responsible for autosomal recessive early‐onset Parkinson Disease.^[^ ^44^ ^]^ Loss of myocardial Park7 protein has been observed in end‐stage heart failure patients^[^ ^19^ ^]^ and Park7 knockout mice were more vulnerable to myocardial infarction and pressure overload challenges.^[^ ^19^ ^]^ Clinically, ability to predict DoIC susceptibility remains lacking.^[^ ^45^ ^]^ The relationship between DOX‐induced Park7 loss and Park7 mutation induced iron accumulation in neurons conveys a possibility that iron overload is more toxic to non‐dividing systems.

In accordance to prior reports of Park7–p53 interaction,^[^ ^21^, ^46^ ^]^ we identified and showed that DOX‐induced p53 binds to Park7 and accelerates its degradation likely through ubiquitination which is in accordance to previous studies where we show that without affecting Park7 mRNA levels, p53 deficient cells exhibit increased Park7 protein levels which is in good agreement with prior observations.^[^ ^21^ ^]^ Although we demonstrated p53 binding to Park7 increases ubiquitination signal of Park7, there are several limitations one would need to consider. First, lack of ubiquitination sites on Park7 limits us to design experiments to demonstrate whether p53 ubiquitinates Park7 directly or indirectly. Second, we did not consider inhibition of p53 transcription activity by Park7 in our system.^[^ ^47^ ^]^ Although it has been demonstrated that p53 can actively recruit E3 ligase Siah1 to drive ubiquitylation‐dependent degradation of telomeric repeat binding factor 2,^[^ ^48^ ^]^ which E3 ligase is recruited by p53 in DOX‐treated cardiomyocytes remain to be explored further.

In DoIC, p53 is activated through oxidative DNA damage‐ataxia telangiectasia mutated (ATM)‐p53 pathway.^[^ ^49^ ^]^ DOX‐induced topoisomerase II*β* inhibition and double‐stranded breaks result in DNA damage and ATM activation.^[^ ^50^ ^]^ One of the most critical downstream targets of ATM is p53, which is activated into a form with improved DNA binding affinity and rapidly accumulates inside of the cell.^[^ ^49^ ^]^ p53 is also identified as the critical transcriptome regulator of DoIC in a study using transcriptomic profiling, with p53‐induced upregulation in death receptors potentially playing a role in increased cardiomyocyte apoptosis.^[^ ^22^ ^]^ Beyond its effects on apoptosis, conflicting evidence of p53 either promoting or suppressing ferroptosis have been reported. On one hand, p53 can promote ferroptosis by downregulating the expression of solute carrier family 7 member 11 (SLC7A11)^[^ ^51^ ^]^ or upregulating the expression of spermidine/spermine N1‐acetyltransferase 1^[^ ^52^ ^]^ and glutaminase 2.^[^ ^53^ ^]^ On the other hand, p53 can suppress ferroptosis through the direct inhibition of dipeptidyl peptidase 4 activity^[^ ^54^ ^]^ or by the induction of cyclin dependent kinase inhibitor 1A/p21 expression.^[^ ^55^ ^]^ However, these aforementioned pathways may be peripheral given that the trigger of cellular ferroptosis is iron overload. Here we demonstrate that the p53–Park7 degradation lies upstream of iron dysregulation and any interceptions to prevent iron accumulation prevents myocardial ferroptosis.

It is important to point out that while a good majority of cancers exhibit p53 mutation that can render p53 null or p53 inactive status,^[^ ^56^ ^]^ p53 too plays an important role in cancer ferroptosis.^[^ ^51^, ^52^, ^54^ ^]^ Recent evidences show that Park7 upregulation confers protection against ferroptosis in cancer cells.^[^ ^20^ ^]^ Thus, together with our data, it is not unexpected to predict that more ferroptotic damage occurs in other healthy organs compared to cancer cells when patients are subjected to DOX treatment. Circumventing the p53–Park7 axis in future chemotherapeutics will help improve safety indexes.

In summary, we show that Park7‐dependent iron removal pathway is important to maintain iron homeostasis in cardiomyocytes and this protective ability is lost in presence of DOX‐induced p53 activation. Although our in vivo rescue experiments showed that restoration of Park7 can greatly ameliorate DoIC, absence of complete reversal is likely due to DOX‐induced topoisomerase toxicity.^[^ ^4^ ^]^ Our study proposes a new perspective on the pathogenesis and treatment of DoIC, and provides a new consideration for designing new chemotherapies.

## 4 Experimental Section

### Animal Experiments

The study was ethically conducted in strict accordance with the recommendations of the “Guide for the Care and Use of Laboratory Animals” and approved by the Animal Experiment Ethics Committee of Shanghai ninth people's hospital [HKDL‐2018‐282]. Wild type male 6 weeks old C57BL/6J mice weighing 18–20 g were purchased from GemPharmatech Co., Ltd (Jiangsu, China) and kept in sterilized filter top cages with controlled humidity and a 12 h day/night cycle at 22 °C. All experiments were started after 1 week of the acclimation period. To generate the chronic DoIC model, mice received a weekly i.p. injection of DOX (5 mg kg^−1^; MedChem Express, USA) for 4 weeks. To inhibit ferroptosis, mice were given a daily i.p. injection of Fer‐1 (a lipid peroxide scavenger; 1 mg kg^−1^; MedChem Express, USA) during the DOX treatment, or a weekly i.p. injection of DFO (an iron chelator; 100 mg kg^−1^; MedChem Express, USA) or DXZ (the only FDA‐approved drug for the prevention and treatment of DoIC, 20 mg kg^−1^; MedChem Express, USA) 1 h before DOX injection. To obtain the cardiomyocyte‐specific p53 knockout mice, p53‐floxed mice were crossed with Myh6‐Cre mice as previously described.^[^ ^30^ ^]^ Age matched p53^f/f^/Cre^−^ and p53^f/f^/Cre^+^ mice were used. To overexpress Park7, 2 weeks prior to DOX induction, mice received a 1 × 10^11^ vg single i.v. injection of adeno‐associated serotype 9 (AAV9) virus carrying cardiac troponin T (cTnT) promoter driven mouse Park7 cDNA (AAV9‐Park7). Empty AAV9‐cTnT served as vector control (AAV9‐Vector) (Genomeditech, China).

### Echocardiography

Transthoracic echocardiography was performed using a Visual Sonics Vevo 3100 system equipped with MS400 transducer (FUJIFILM Visual Sonics, Japan). Mice were anesthetized and maintained under 1–2% isoflurane and 2 L min^−1^ 20% oxygen during the procedure. Echocardiographic measurements were performed by a blinded investigator as previously described.^[^ ^57^ ^]^ LVEF, LVFS, and GLS were analyzed using Vevo Analysis software (FUJIFILM Visual Sonics, Japan).

### Histological Evaluation

Hematoxylin‐Eosin (HE) and Masson's trichrome staining were performed. Murine hearts were fixed for 24 h in 4% paraformaldehyde at 4 °C, paraffin embedded, and transversely sectioned. Sections (5 µm) were deparaffinized and rehydrated by gradient elution using xylene and ethanol, and were stained by hematoxylin‐eosin (Servicebio Biotech, China) and Masson trichrome staining reagent (Servicebio Biotech, China) per manufacturer's instructions.

### Measurement of Iron Content and MDA Levels

The MDA levels in plasma and heart tissues were determined using the Colorimetric MDA Assay Kit (Jiancheng, China), and the Fe^2+^ and Fe^3+^ levels in heart tissues were determined by Iron Assay Kit (DOJINDO, Japan) per manufacturers’ instructions.

### AMCMs and NMCMs Isolation

For AMCMs, mice were heparinized and anesthetized using deep isoflurane (5%) anesthesia. The hearts were surgically isolated, washed in ice‐cold cardiomyocyte isolation buffer (CIB; 120 mm NaCl, 5.4 mm KCl, 0.5 mm MgSO4, 0.33 mm NaH2PO4, 25 mm NaHCO3, 22 mm glucose, 25 mm HEPES, 10 mm BDM, and 30 mm taurine), cannulated via the ascending aorta and digested through perfusion with an enzyme CIB buffer containing 1 mg mL^−1^ Type II Collagenase (BioFroxx, Germany) and 0.6 mg mL^−1^ Type IV Collagenase (BioFroxx, Germany) for 15 min at 37 °C using a Langendorff perfusion system. The digested hearts were mechanically shredded and dissociated in Minimum Essential Medium Eagle (Sigma‐Aldrich, USA) supplemented with 10% bovine serum albumin (BSA; Sigma‐Aldrich, USA). After filtration with a 100 µm mesh filter and centrifugation at 500 rpm for 5 min, harvested AMCMs were reintroduced with calcium in four concentration gradients, ranging from 0 to 900 µm.

For NMCMs, newborn C57BL/6J mice (within 72 h) were euthanized by decapitation and the hearts were collected. NMCMs were isolated as previously described^[^ ^57^ ^]^ and identified by immunofluorescence of cTnT.

### Cell Culture and Transfection

NMCMs were media changed every 3 days until autonomous contractions appeared. DOX (1 µm, 24 h) treatment was used to establish a DoIC model in vitro. To inhibit ferroptosis, Fer‐1 (2 µm, 24 h), DFO (5 µm, 24 h), or DXZ (1 µm, 24 h) were used prior to DOX treatment. FAC (5 mm, 24 h; MedChem Express, USA) was used to induce secondary iron overload in NMCMs as previous described.^[^ ^24^ ^]^ MG132 (20 µm; MedChem Express, USA) and CHX (100 µm; MedChem Express, USA) time course treatment were used for protein half‐life determination. To evaluate p53 involvement in ferroptosis activation, Nutlin‐3a (3 µm, 24 h) was to induce p53 activation in NMCMs. NMCMs isolated from p53^f/f^/Cre^−^ and p53^f/f^/Cre^+^ mice were used as a p53 knockout validation. A lentivirus vector (Lenti‐CMV‐PGK‐puro) carrying mouse Park7 cDNA (Lenti‐Park7) or Park7 shRNA (Lenti‐shPark7) (Genomeditech, China) was used for overexpression or knockdown of Park7. NMCMs were treated with corresponding viruses 24 h prior to DOX treatment. The target sequences are listed in Table S1, Supporting Information.

### Cardiomyocyte Contractility Assay

Contractility of AMCMs were evaluated using an IonOptix System (IonOptix, USA).^[^ ^30^ ^]^ Briefly, Langendorff isolated AMCMs were seeded onto laminin (Sigma‐Aldrich, USA)‐coated imaging dishes in DMEM supplemented with 10% FBS and incubated for 30 min at 37 °C. The cells were field stimulated (10 V) at a frequency of 1 Hz, and the changes of sarcomere length were simultaneously recorded and calculated.

For cultured NMCMs, spontaneous contractions were recorded using an Olympus IX83 inverted microscope (Olympus, Japan) at 50 fps. The contraction speeds were determined using a Matlab‐based motion‐tracking software (MathWorks, USA) as previously described.^[^ ^58^ ^]^

### Live Cell Imaging

In AMCMs or NMCMs, cellular or mitochondrial iron levels were determined by FerroOrange staining (1 µm, DOJINDO, Japan) or Mito‐FerroGreen staining (5 µm, DOJINDO, Japan); cellular or mitochondrial lipid peroxides were determined by Liperfluo staining (5 µm, DOJINDO, Japan) or MitoPeDPP staining (0.5 µm, DOJINDO, Japan); cell ROS level was determined by CellROS staining (1 µm, Sigma‐Aldrich, USA) and mitochondrial superoxide level was determined by MitoSox staining (1 µm, Invitrogen, USA). Mitochondrial membrane potential was measured using JC‐1 staining (1 µm, Invitrogen, USA) for NMCMs and TMRM staining (1 µm, Invitrogen, USA) for AMCMs. Hoechst 33 342 were used for nuclei staining. All living cell dyes were used per manufacturer's instructions. Fluorescence intensities were captured and quantified using an Operetta CLS High Content Imaging System (Perkin Elmer, USA) or BD FACS Celesta flow cytometry (BD Biosciences, USA). All results were presented as relative intensity using vehicle group for normalization.

### Cell Viability Assay

Cell viabilities of NMCMs were measured using propidium iodide (PI) staining (DOJINDO, Japan). Treated NMCMs were digested using trypsin and washed twice with PBS, followed by PI staining for 15 min. PI positive NMCMs were analyzed and calculated by BD FACSCelesta flow cytometry (BD Biosciences, USA).

### Oxygen Consumption Measurements

Oxygen consumption rates of NMCMs were measured using on a Seahorse XFe96 Extracellular Flux Analyzer in conjunction with Seahorse Cell Mito Stress Test Kit (Agilent, USA). Cells were seeded on Seahorse cell culture plates and treated according to aforementioned protocol.^[^ ^30^ ^]^ The completed culture media were changed to Seahorse XF DMEM supplemented with 5 µm glucose, 1 µm pyruvate, and 10 µm glutamine 1 h prior to measurement. After 1 h incubation at 37 °C in CO_2_‐free incubator, oxygen consumption rates were measured in basal, oligomycin (1.5 m), FCCP (1 m), and Rotenone/Antimycin A (0.5 m) environments. Oxygen consumption rates were normalized to total cell number determined by Hoechst 33 342 staining which was quantified at the end of the Seahorse experiment.

### Measurement of Aconitase Activity

Cellular aconitase activity and mitochondrial aconitase activity were evaluated using an aconitase activity kit (Sigma‐Aldrich, USA) per manufacturer's protocol. Briefly, AMCMs or NMCMs were homogenized in assay buffer with a brief sonication and differentially centrifuged to obtain the mitochondrial and cytosolic subcellular fractions. The lysates were treated with aconitase activation solution, mixed with a 1:1 cysteine and NH_4_Fe(SO_4_)_2_ solution on ice for 1 h, and incubated with reaction mix containing assay buffer, enzyme mix, and substrate at 25 °C for 45 min. Next, samples were incubated with the developer solution for 10 min at 25 °C and 450 nm absorbances were measured using a 96‐well microplate reader (Bio‐Tek, USA). Protein content of the samples were measured using the bicinchoninic acid method (BCA; Beyotime Biotechnology, China). Aconitase activity was calculated as milliunit per milligram protein.

### Protein Extraction and Western Blot

Protein extracts were isolated from AMCMs and NMCMs at 4 °C using radioimmunoprecipitation buffer with protease and phosphatase inhibitor cocktail. Lysed cells were centrifugated at 12 000 rpm for 15 min at 4 °C and protein lysates were transferred to a new Eppendorf tube. The protein concentration was determined using the BCA method (Beyotime Biotechnology, China) and analyzed using Western immunoblot as previously described.^[^ ^57^ ^]^ All primary and secondary antibodies were shown in Table S2, Supporting Information. The results were digitally imaged and analyzed with the Odyssey Infrared Imaging System (LICOR, USA).

### Mass Spectrometry Analysis

The protein samples from Vehicle, DOX, and FAC treated NMCMs were isolated using EasyPep MS lysis solution (Thermo Fisher scientific, USA). The protein concentration was quantified using the BCA method. The eluted proteins were analyzed using a Q Exactive HF Orbitrap Mass Spectrometer (Thermo Fisher Scientific, USA). Proteins of a 1.2‐fold change (≥1.2 or ≤0.83) of Coverage with or without *P* < 0.05 cutoff were included into subsequent Gene Ontology/DAVID analyses (http://david.abcc.ncifcrf.gov).

### Co‐IP

NMCMs were collected and lysed in immunoprecipitation lysis buffer (Beyotime Biotechnology, China) containing proteinase cocktail with a brief sonication. The protein concentration was determined using the BCA method. Cell lysates were incubated with Park7 or p53 antibody (1 µg mg^−1^ protein; Table S2, Supporting Information) overnight at 4 °C, followed by incubating with 30 µL BSA blocked Protein A/G magnetic beads (Bimake, USA) for 6 h at 4 °C. The isotype IgG antibody was used as negative control. The precipitated beads were washed three times with ice‐cold immunoprecipitation lysis buffer and proteins were eluted in SDS loading buffer (Beyotime Biotechnology, China) by incubating at 95 °C for 10 min. The eluted proteins were analyzed by immunoblotting.

### RNA Preparation and Quantitative Real‐Time PCR

Total RNA was isolated using TRIzol regent (Invitrogen, USA) and reverse‐transcribed using HiScriptII Reverse Transcriptase kit (Vazyme, China) per manufacturer's instructions. Transcripts of interest were amplified and quantified in triplicate using an Applied Biosystems 6Flex system (Applied Biosystems, USA) with SYBR Green Master Mix (Bimake, USA). The fold difference of gene expression was calculated using the 2^−∆∆ct^ method and presented relative to Gapdh mRNA. All primers used can be found in Table S3, Supporting Information.

### Immunofluorescence Staining

Heart sections were deparaffinized and rehydrated by gradient elution using xylene and ethanol, followed by PBS washes. Antigen retrieval was performed in Tris‐EDTA solution for 20 min at 95 °C. For NMCMs, cells were rinsed with PBS, fixed with 4% paraformaldehyde for 20 min at room temperature. Prepared heart sections and NMCMs were blocked by incubation with PBS containing 10% goat serum (Beyotime Biotechnology, China) and 0.3% Triton‐100 (Sigma‐Aldrich, USA) for 1 h at 37 °C, and incubated with primary antibodies (Table S2, Supporting Information) overnight at 4 °C. Slides were washed three times and incubated with Alexa Fluro conjugated secondary antibody (Table S2, Supporting Information) for 1 h at room temperature. Nuclei were stained using 4′,6‐diamidino‐2‐phenylindole for 10 min. Image acquisition was performed using a Zeiss LSM880 confocal microscope and analyzed using ZEN software (Zeiss, Germany).

### Transmission Electron Microscopy

For transmission electron microscopy, freshly excised heart tissues were immediately fixed by immersion into 2.5% glutaraldehyde at 4 °C overnight. The fixed samples were washed three times with 0.1 m cacodylate trihydrate buffer and post‐fixed with 1% osmium tetroxide for 1 h. After 3 PB buffer washes, the samples were dehydrated in ethanol gradients, incubated with acetone, and embedded in ethoxyline resin. Ultrathin sections were assembled to a copper grid and the images were obtained using a FEI (Tecnai G2 Spirit 120 kV) electron microscope (FEI Italia, Italy).

### Statistics Analysis

Data were analyzed and graphed using GraphPad Prism software (Version 9.1.1). All continuous variables were presented as mean ± SD with minimum of six mice for in vivo experiments and three independent experiments for in vitro experiments. For comparisons between two groups, two‐tailed Student's *t*‐tests were used. For comparisons among multiple groups, *t* one‐way ANOVA followed by Tukey's post hoc test was used. A *P* value <0.05 was considered statistically significant. The statistical analyses and sample size applied for each experiment were indicated in the figure legends.

## Conflict of Interest

The authors declare no conflict of interest.

## Author Contributions

J.P. and W.X. contributed equally to this work. J.P., A.C.Y.C., and C.W. conceived and designed this project. J.G. and C.W. provided reagents. J.P., W.X., A.Z., H.L., J.K., and S.H. performed the in vivo experiments. J.P., H.Z., and L.G. performed the in vitro experiments. J.P. and J.Z. analyzed data and drafted the manuscript. A.C.Y.C., J.G., and C.W. revised the initial draft. All authors provided critical discussions, proof‐read the draft, and gave final approval of the manuscript.

## Supporting information

Supporting Information

## Data Availability

The data that support the findings of this study are available from the corresponding author upon reasonable request.
