# Supplementary material for: The Imbalance of p53–Park7 Signaling Axis Induces Iron Homeostasis Dysfunction in Doxorubicin‐Challenged Cardiomyocytes
Source: Adv Sci (Weinh). 2023 Mar 26;10(15):2206007. doi: 10.1002/advs.202206007 (PMC10214246; doi:10.1002/advs.202206007)
Supplement: Supplementary file 1 — Supporting Information [file ADVS-10-2206007-s001.pdf]

ADVANCED SCIENCE
Open Access

Supporting Information

for *Adv. Sci.*, DOI 10.1002/advs.202206007

# The Imbalance of p53–Park7 Signaling Axis Induces Iron Homeostasis Dysfunction in Doxorubicin-Challenged Cardiomyocytes

*Jianan Pan, Weiyao Xiong, Alian Zhang, Hui Zhang, Hao Lin, Lin Gao, Jiahan Ke, Shuying Huang, Junfeng Zhang, Jun Gu*, Alex Chia Yu Chang* and Changqian Wang**

**Supporting Information**

# The Imbalance of p53-Park7 Signaling Axis Induces Iron Homeostasis Dysfunction in Doxorubicin-Challenged Cardiomyocytes

**Table S1 Sequences of gene-shRNA**

| **shRNA** | **Sequence** |
|---|---|
| sh*Park7*-1 | GCAGTGTAGCCGTGATGTAAT |
| sh*Park7*-2 | GAAATCTGGGTGCACAGAATT |
| sh*Park7*-3 | GCTCTGTTGGCTCACGAAGTA |
| Scramble | UUCUCCGAACGUGUCACGUTT |

**Table S2 Antibodies.**

| **Antibodies** | **Cat. No. and Company** | **Dilution Ratio** |
|---|---|---|
| cTnT | ab8295, Abcam | 1:200 for IF |
| TfR | ab214039, Abcam | 1:1000 for WB |
| FPN | 26601-1-AP, Proteintech | 1:1000 for WB |
| FTH | sc-376594, Santa Cruz Biotechnology | 1:500 for WB |
| IRP1 | sc-166022, Santa Cruz Biotechnology | 1:500 for WB |
| IRP2 | 23829-1-AP, Proteintech | 1:1000 for WB |
| MFRN | 26469-1-AP, Proteintech | 1:1000 for WB |
| Park7 | 5933, Cell Signaling Technology | 1:1000 for WB; 1:100 for IF; 1μg/mg protein for IP |
| p53 | 2524, Cell Signaling Technology | 1:1000 for WB; 1μg/mg protein for IP |

| | | |
|---|---|---|
| p53 | 10442-1-AP, Proteintech | 1:1000 for WB |
| GAPDH | 60004-1-Ig, Proteintech | 1:10000 for WB |
| HSP60 | 15282-1-AP, Proteintech | 1:10000 for WB |
| Normal Rabbit IgG | 2729, Cell Signaling Technology | 1μg/mg protein for IP |
| Normal Mouse lgG | B900620, Proteintech | 1μg/mg protein for IP |
| Anti-mouse IgG (H+L) (DyLight™ 800 4X PEG Conjugate) | 5257, Cell Signaling Technology | 1:30000 for WB |
| Anti-rabbit IgG (H+L) (DyLight™ 800 4X PEG Conjugate) | 5151, Cell Signaling Technology | 1:30000 for WB |
| Anti-rabbit IgG (H+L), F(ab')2 Fragment (Alexa Fluor® 594 Conjugate) | 8889, Cell Signaling Technology | 1:1000 for IF |

| | | |
|---|---|---|
| Anti-mouse IgG (H+L), F(ab')2 Fragment (Alexa Fluor® 488 Conjugate) | 4408, Cell Signaling Technology | 1:1000 for IF |
| Anti-mouse IgG (H+L), F(ab')2 Fragment (Alexa Fluor® 594 Conjugate) | 8890, Cell Signaling Technology | 1:1000 for IF |

**Table S3 Primers for real-time PCR.**

| **Primers** | **Sequences 5'---3'** |
|---|---|
| Mouse *Ptgs2* Fw | CTGCGCCTTTTCAAGGATGG |
| Mouse *Ptgs2* Re | GGGGATACACCTCTCCACCA |
| Mouse *Park7* Fw | AGTCGCCTATGGTGAAGGAGATCC |
| Mouse *Park7* Re | TGAGCCAACAGAGCCGTAGGAC |
| Mouse *p53* Fw | AGTAAAGGCTCTAAAGCTCACCC |
| Mouse *p53* Re | CCCATCGTCAACTTGGTCCA |
| Mouse *Gapdh* Fw | ATCATCCCTGCATCCACT |
| Mouse *Gapdh* Re | ATCCACGACGGACACATT |

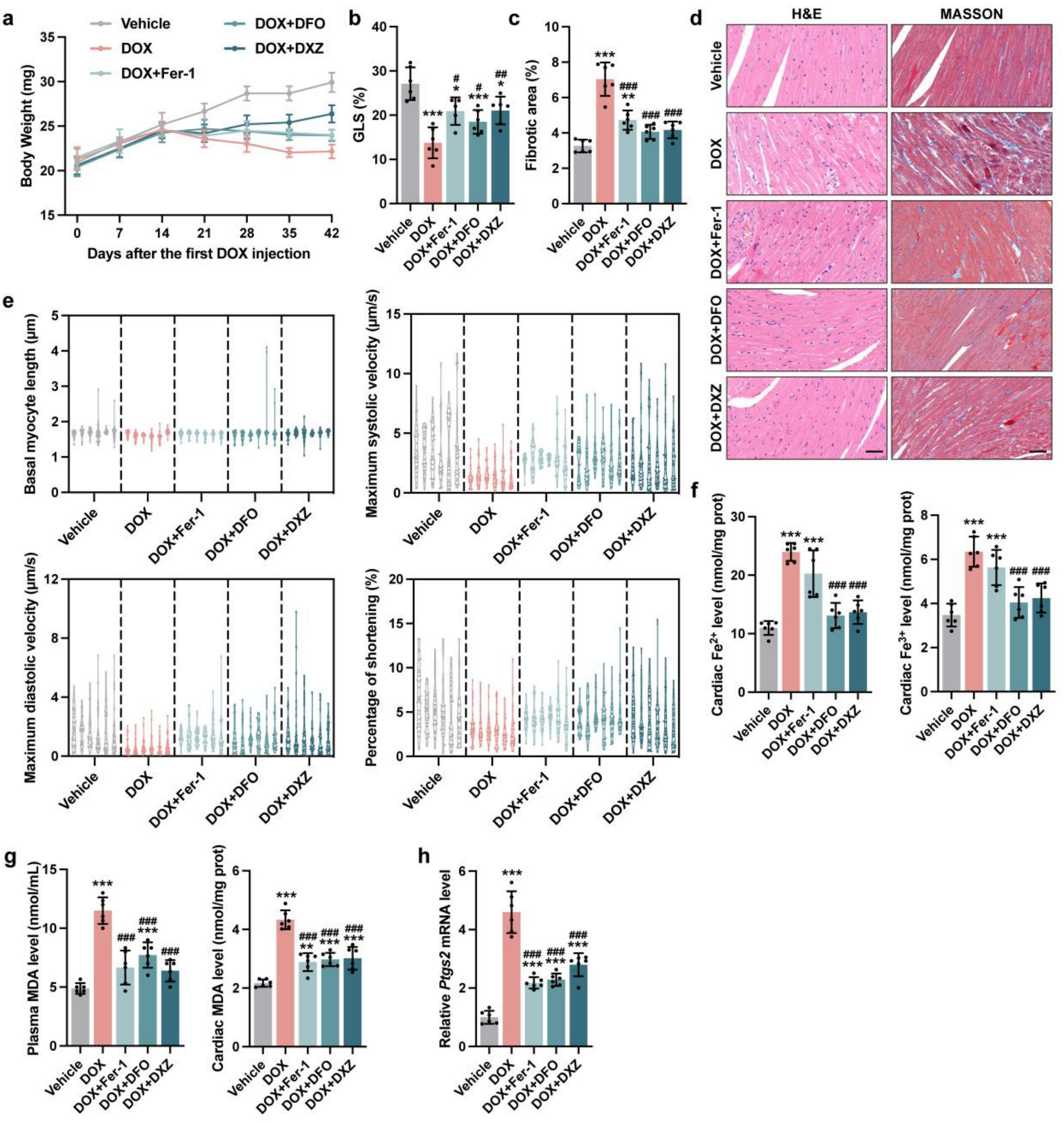


**Figure S1 Blocking myocardial ferroptosis significantly alleviates DoIC *in vivo*.**

**a)** Body weight tracing during DoIC model (n = 10). **b)** Quantification of global longitudinal strain (GLS; n=6). **c)** Percentage of fibrotic area in heart sections (n = 6). **d)** Representative micrographs of H&E and Masson's trichrome staining in heart sections (scale bars, 50 μm). **e)** Calculation of basal myocyte length, maximum systolic velocity, maximum diastolic velocity and percentage of shortening of AMCMs of each mouse (n = 30 AMCMs per mouse). **f)** $Fe^{2+}$

and $Fe^{3+}$ levels in heart tissues (n = 6). **g)** MDA level in plasma and heart tissues (n = 6). **h)** *Ptgs2* mRNA expression in AMCMs (n = 6). The data are expressed as mean ± SD and analyzed using one-way ANOVA followed by Tukey's post hoc test, *$p < 0.05$, **$p < 0.01$ and ***$p < 0.001$ vs. Vehicle group; #$p < 0.05$, ##$p < 0.01$ and ###$p < 0.001$ vs. DOX group.

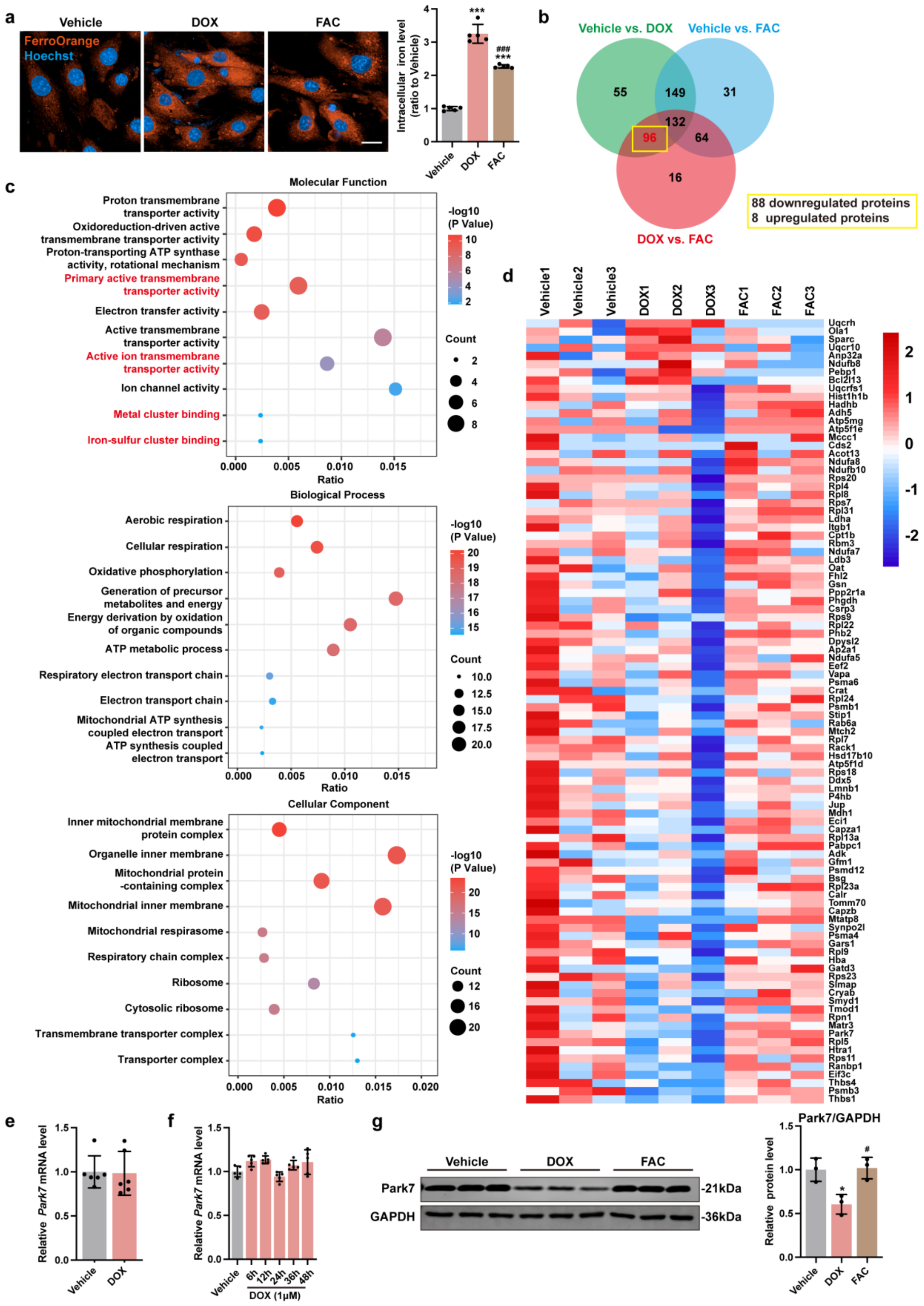


**Figure S2 DOX impairs iron homeostasis in cardiomyocytes which is associated with**

**Park7 downregulation.**

**a)** Representative micrographs and quantitative analysis of intracellular iron level (FerroOrange staining) (scale bars, 20 μm; n = 5). **b)** Venn analysis of differentially expressed proteins in NMCMs (Proteins of a 1.2-fold change (≥1.2 or ≤0.83) of Coverage without $P < 0.05$ cutoff). **c)** Gene ontology analysis of differentially expressed proteins. **d)** Heatmap of DOX-dysregulated proteins. **e)** *Park7* mRNA expression in AMCMs from chronic mice DoIC model (n = 6). **f)** *Park7* mRNA expression in NMCMs treated with 1 μM DOX in different times (n = 5). g) Representative immunoblots and quantitative analysis of Park7 in NMCMs treated with DOX or FAC (n = 3). The data are expressed as mean ± SD and analyzed using Student's t-test and one-way ANOVA followed by Tukey's post hoc test, ***$p < 0.001$ vs. Vehicle group; ###$p < 0.001$ vs. DOX group.

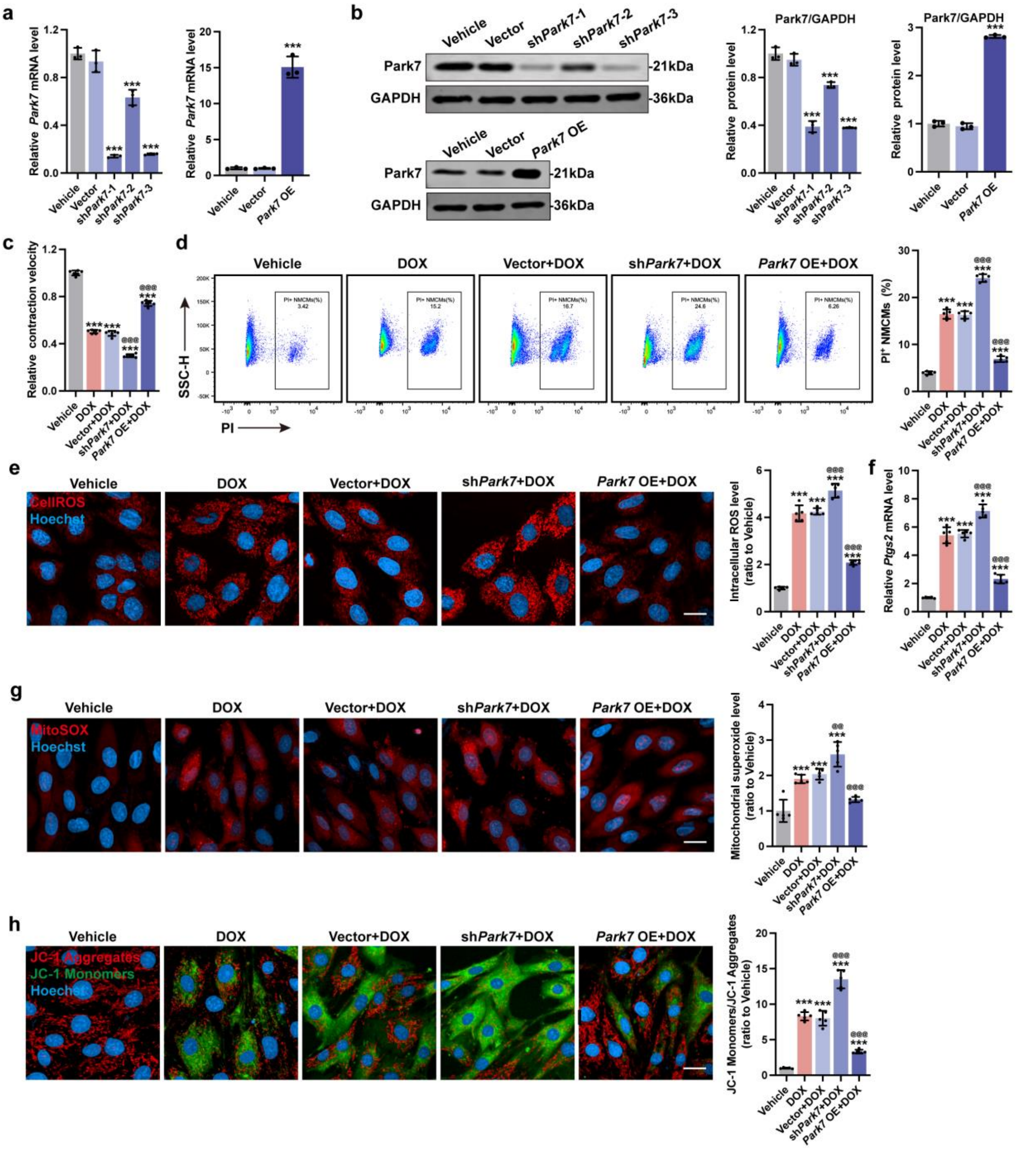


**Figure S3 DOX-induced downregulation of Park7 results in loss of iron clearance and promotes mitochondrial iron overload in cardiomyocytes.**

**a)** The infection efficiencies for downregulation or overexpression of *Park7* in NMCMs in mRNA level (n = 5). **b)** The infection efficiencies for downregulation or overexpression of *Park7* in NMCMs in protein level (n = 3). **c)** Contractile velocity of NMCMs extrapolated from live cell imaging (n = 6). **d)** The percentage of $PI^+$ NMCMs were calculated using flow

cytometry (n = 5). **e)** Representative micrographs and quantification of intracellular ROS (CellROS staining) in NMCMs (scale bars, 20μm; n = 5). **f)** *Ptgs2* mRNA expression level in NMCMs (n = 5). **g)** Representative micrographs and quantification of mitochondrial superoxide level (MitoSOX staining) in NMCMs (scale bars, 20μm; n=5). **h)** Representative micrographs and quantification of mitochondrial membrane potential (JC-1 staining) in NMCMs (scale bars, 20μm; n = 5). The data are expressed as mean ± SD and analyzed using one-way ANOVA followed by Tukey's post hoc test, ***p < 0.001 vs. Vehicle group; @@p < 0.01 and @@@p < 0.001 vs. Vector+DOX group.

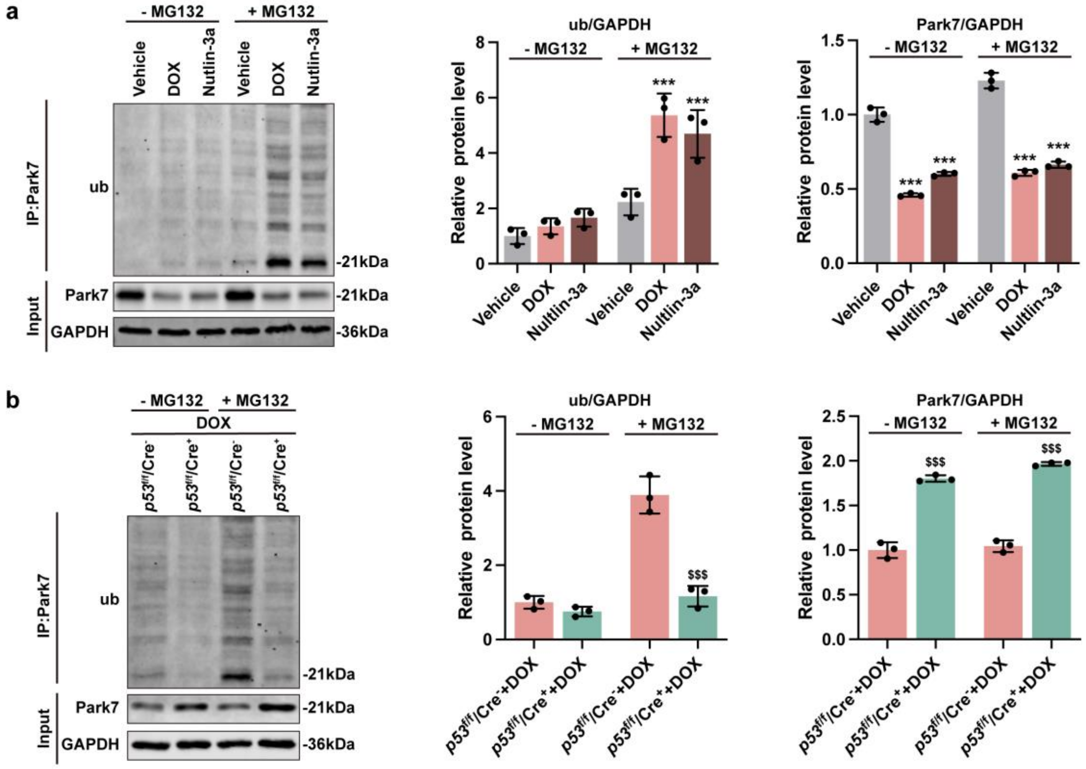


**Figure S4 p53 downregulates Park7 protein levels through ubiquitination.**

**a)** MNCMs were treated by DOX or Nutlin-3a with or without MG132 and Park7 was

isolated by IP. Representative immunoblots and quantitative analysis of Park7 ubiquitination were assessed with anti-ub antibody (n = 3). **b)** MNCMs isolated from *p53*$^{f/f}$/Cre$^{-}$ and *p53*$^{f/f}$/Cre$^{+}$ mice were treated by DOX with or without MG132 and Park7 was isolated by IP. Representative immunoblots and quantitative analysis of Park7 ubiquitination were assessed with anti-ub antibody (n = 3). The data are expressed as mean ± SD and analyzed using one-way ANOVA followed by Tukey's post hoc test, ***$p < 0.001$ vs. Vehicle group under the same condition; $$$$p < 0.001$ vs. *p53*$^{f/f}$/Cre$^{-}$+DOX group under the same condition.

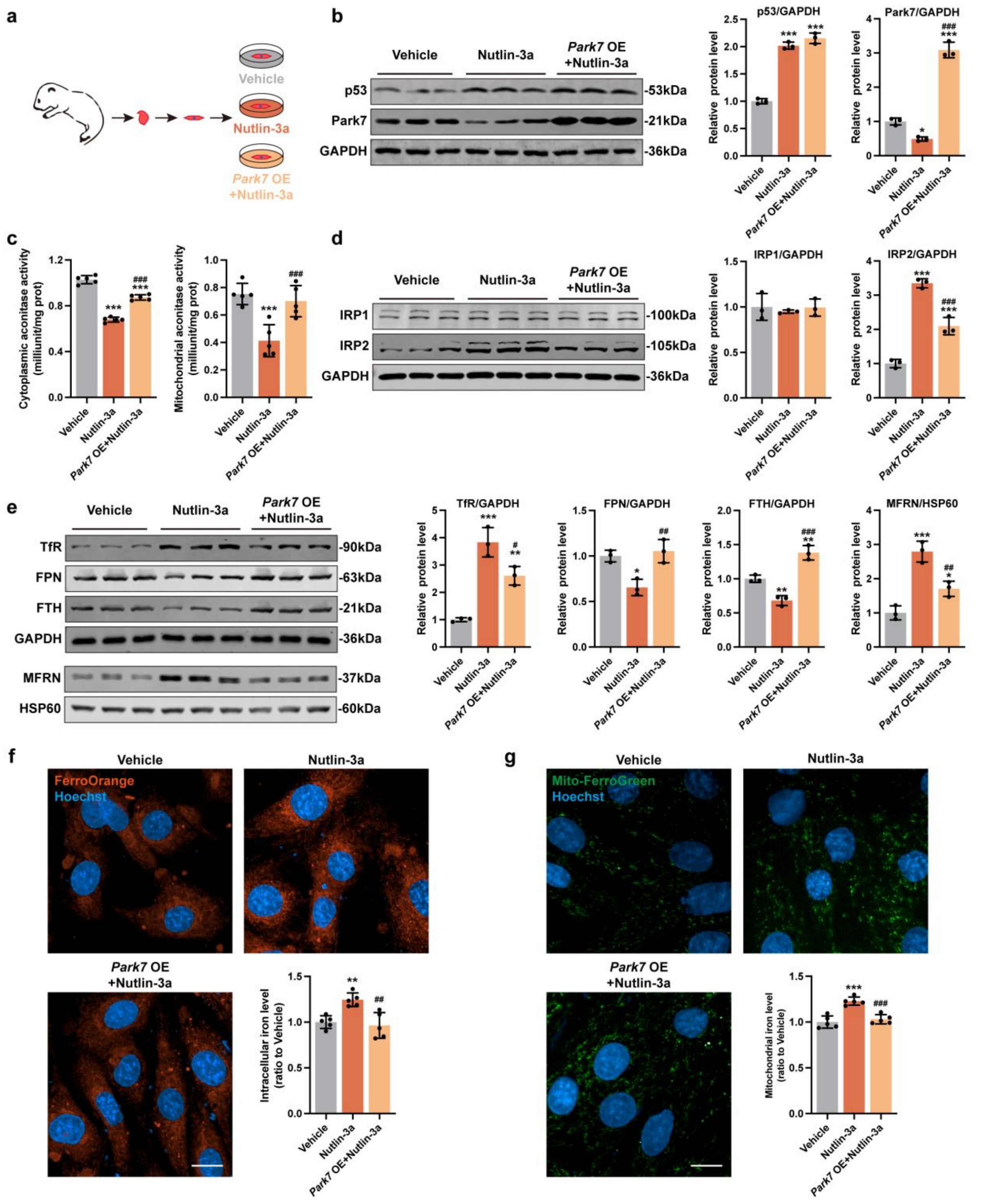


**Figure S5 p53/Park7 axis regulates iron homeostasis in healthy cardiomycytes.**

**a)** Experiment design for p53 activation with or without Park7 rescue in healthy NMCMs. **b)** Representative immunoblots and quantitative analysis of Park7 and p53 protein level (n = 3).

**c)** The cellular aconitase activity and mitochondrial aconitase activity in NMCMs (n = 5). **d)** Representative immunoblots and quantitative analysis of IRP1 and IRP2 protein level in NMCMs (n = 3). **e)** Representative immunoblots and quantitative analysis of iron homeostasis-related proteins in NMCMs (n = 3). **f)** Representative micrographs and quantification of intracellular iron level (FerroOrange staining) in NMCMs (scale bars, 20μm; n = 5). **g)** Representative micrographs and quantification of mitochondrial iron level (Mito-FerroGreen staining) in NMCMs (scale bars, 20μm; n = 5). The data are expressed as mean ± SD and analyzed using one-way ANOVA followed by Tukey's post hoc test, *$p < 0.05$, **$p < 0.01$ and ***$p < 0.001$ vs. Vehicle group; ##$p < 0.01$ and ###$p < 0.001$ vs. Nutlin-3a group.

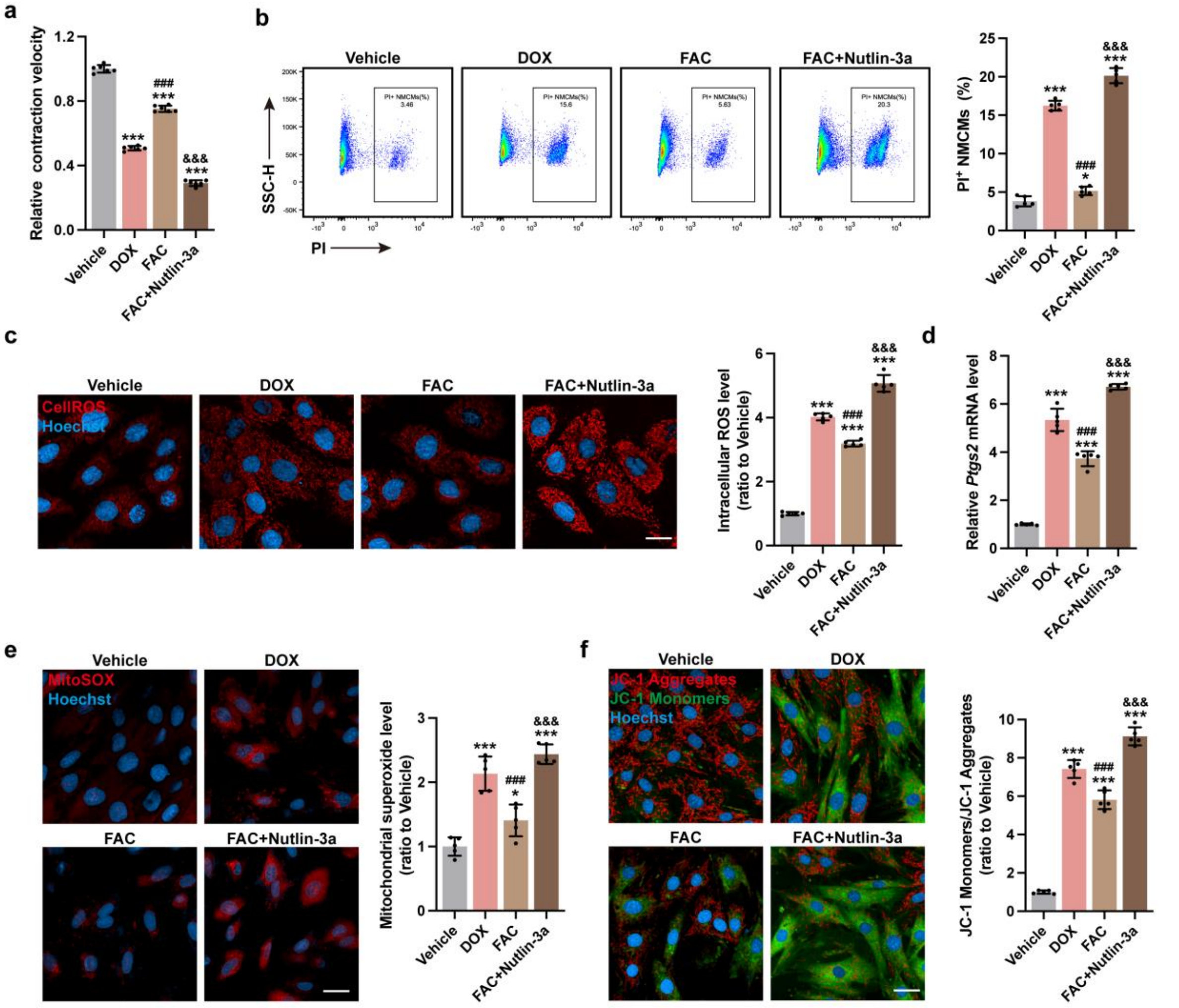


**Figure S6 p53 activation recapitulates ferroptosis phenotype in FAC-treated NMCMs.**

**a)** Contractile velocity of NMCMs extrapolated from live cell imaging (n = 6). **b)** The percentage of $PI^+$ NMCMs were calculated using flow cytometry (n = 5). **c)** Representative micrographs and quantification of intracellular ROS (CellROS staining) in NMCMs (scale bars, 20μm; n = 5). **d)** *Ptgs2* mRNA expression level in NMCMs (n = 5). **e)** Representative micrographs and quantification of mitochondrial superoxide level (MitoSOX staining) in NMCMs (scale bars, 20μm; n = 5). **f)** Representative micrographs and quantification of mitochondrial membrane potential (JC-1 staining) in NMCMs (scale bars, 20μm; n = 5). The

data are expressed as mean ± SD and analyzed using one-way ANOVA followed by Tukey's post hoc test, *p < 0.05 and ***p < 0.001 vs. Vehicle group; ###p < 0.001 vs. DOX group; &&&p < 0.001 vs. FAC group.

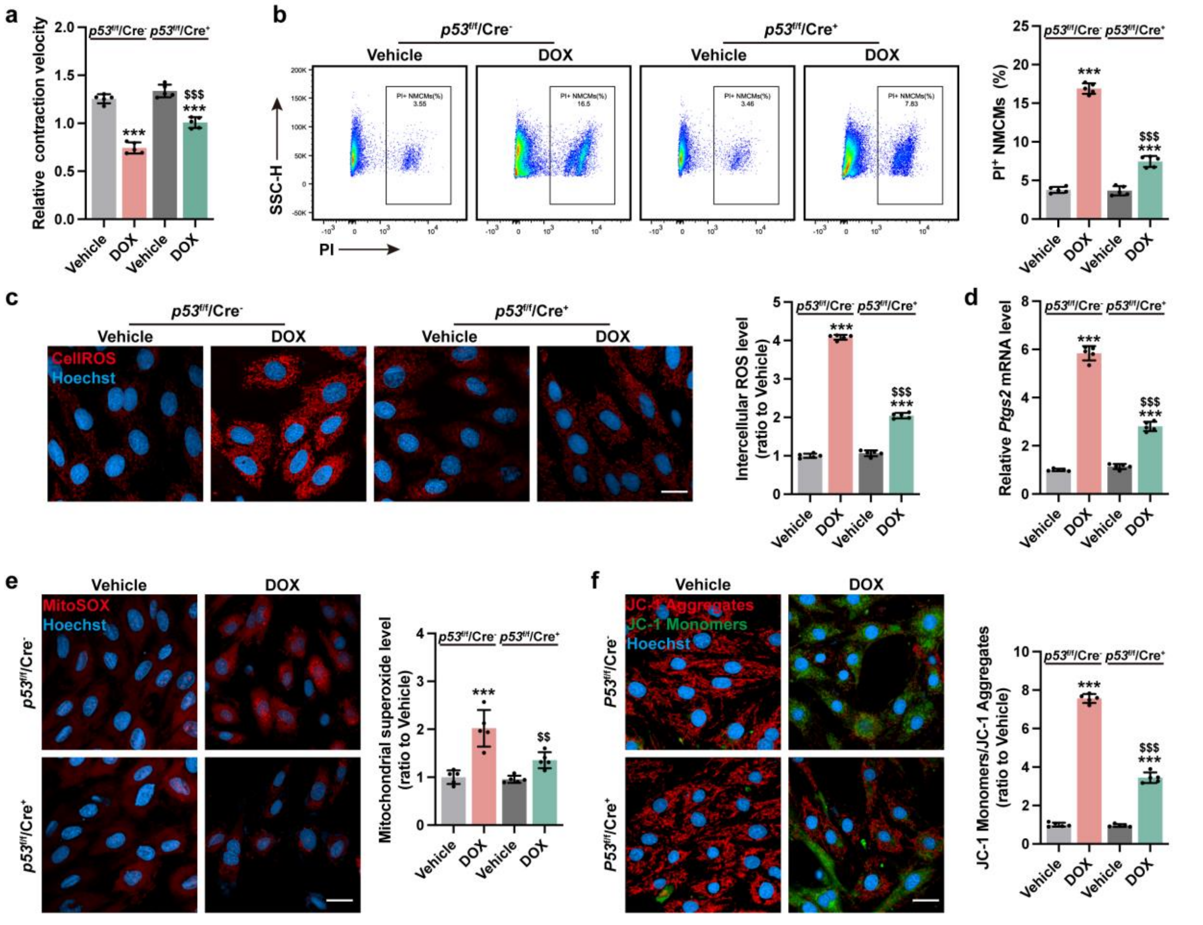


**Figure S7 Knockout of *p53* ameliorated DOX-induced iron metabolism dysfunction and ferroptosis in NMCMs.**

**a)** Contractile velocity of NMCMs extrapolated from live cell imaging (n = 6). **b)** The percentage of $PI^+$ NMCMs were calculated using flow cytometry (n = 5). **c)** Representative micrographs and quantification of intracellular ROS (CellROS staining) in NMCMs (scale bars, 20μm; n = 5). **d)** *Ptgs2* mRNA expression level in NMCMs (n = 5). **e)** Representative

micrographs and quantification of mitochondrial superoxide level (MitoSOX staining) in NMCMs (scale bars, 20μm; n = 5). **f)** Representative micrographs and quantification of mitochondrial membrane potential (JC-1 staining) in NMCMs (scale bars, 20μm; n = 5). The data are expressed as mean ± SD and analyzed using one-way ANOVA followed by Tukey's post hoc test, ***$p < 0.001$ vs. Vehicle group; \$\$$p < 0.01$ and \$\$\$$p < 0.001$ vs. *p53*$^{f/f}$/Cre$^{-}$+DOX group.

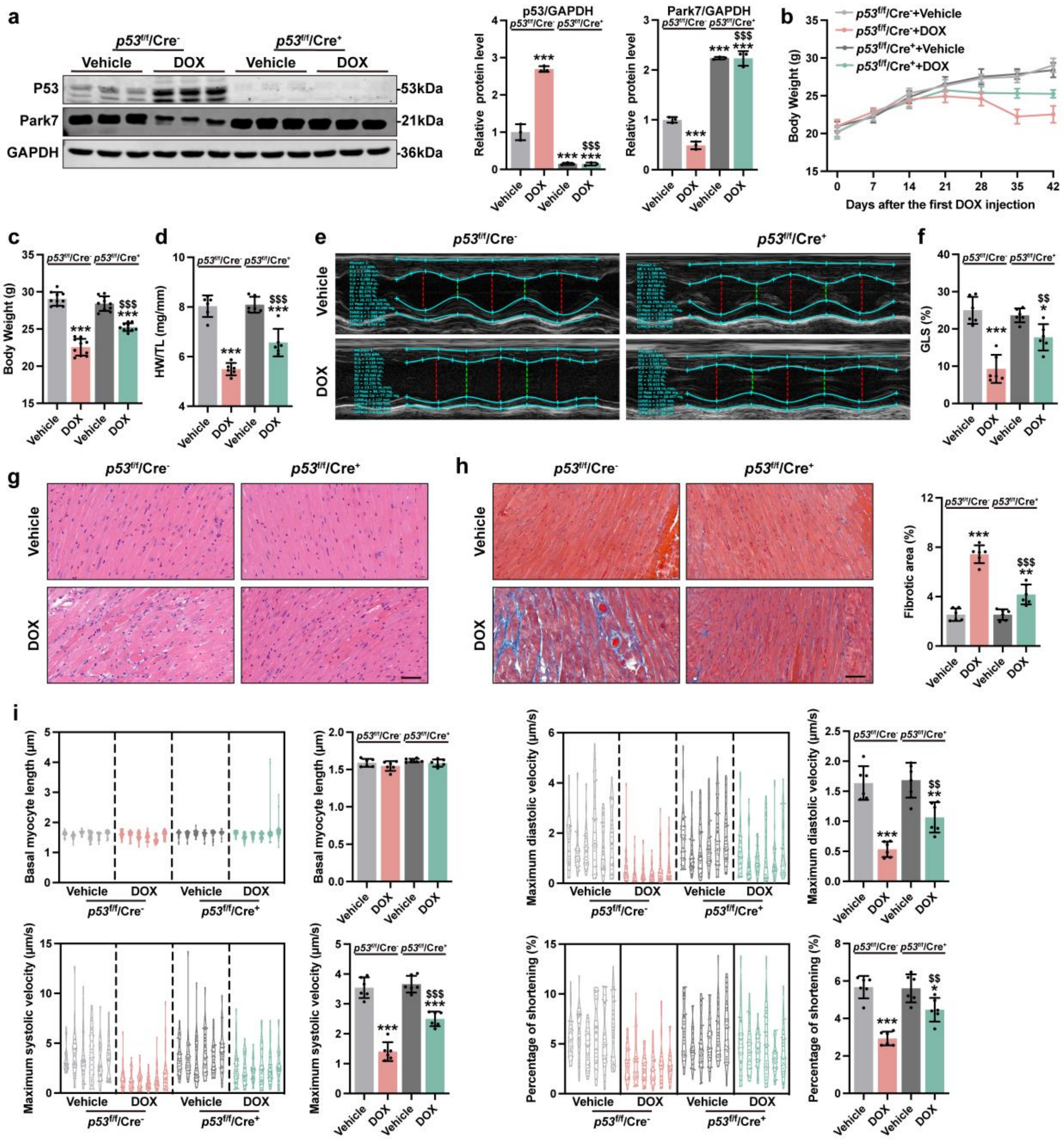


**Figure S8 Knockout of *p53* ameliorates DoIC in mice.**

**a)** Representative immunoblots and quantitative analysis of Park7 and p53 protein level in AMCMs (n = 3). **b)** Body weight tracing during DoIC model (n = 10). **c)** Body weight of mice before sacrificed (n = 10). **d)** Ratio of heart weight to tibia length (HW/TL; n = 6). **e)** Representative M-mode images of transthoracic echocardiography**.** **f)** Quantification of

global longitudinal strain (GLS; n = 6). **g)** Representative micrographs of H&E staining in heart sections (scale bars, 50 μm). **h)** Representative micrographs of Masson's trichrome staining and percentage of fibrotic area in heart sections (scale bars, 50 μm; n = 6). **i)** Calculation of basal myocyte length, maximum systolic velocity, maximum diastolic velocity and percentage of shortening of AMCMs (n = 30 AMCMs per mice, 6mice per group). The data are expressed as mean ± SD and analyzed using one-way ANOVA followed by Tukey's post hoc test, $*p < 0.05$, $**p < 0.01$ and $***p < 0.001$ vs. Vehicle group; $\$\$p < 0.01$ and $\$\$\$p < 0.001$ vs. *p53*$^{f/f}$/Cre$^{-}$+DOX group.

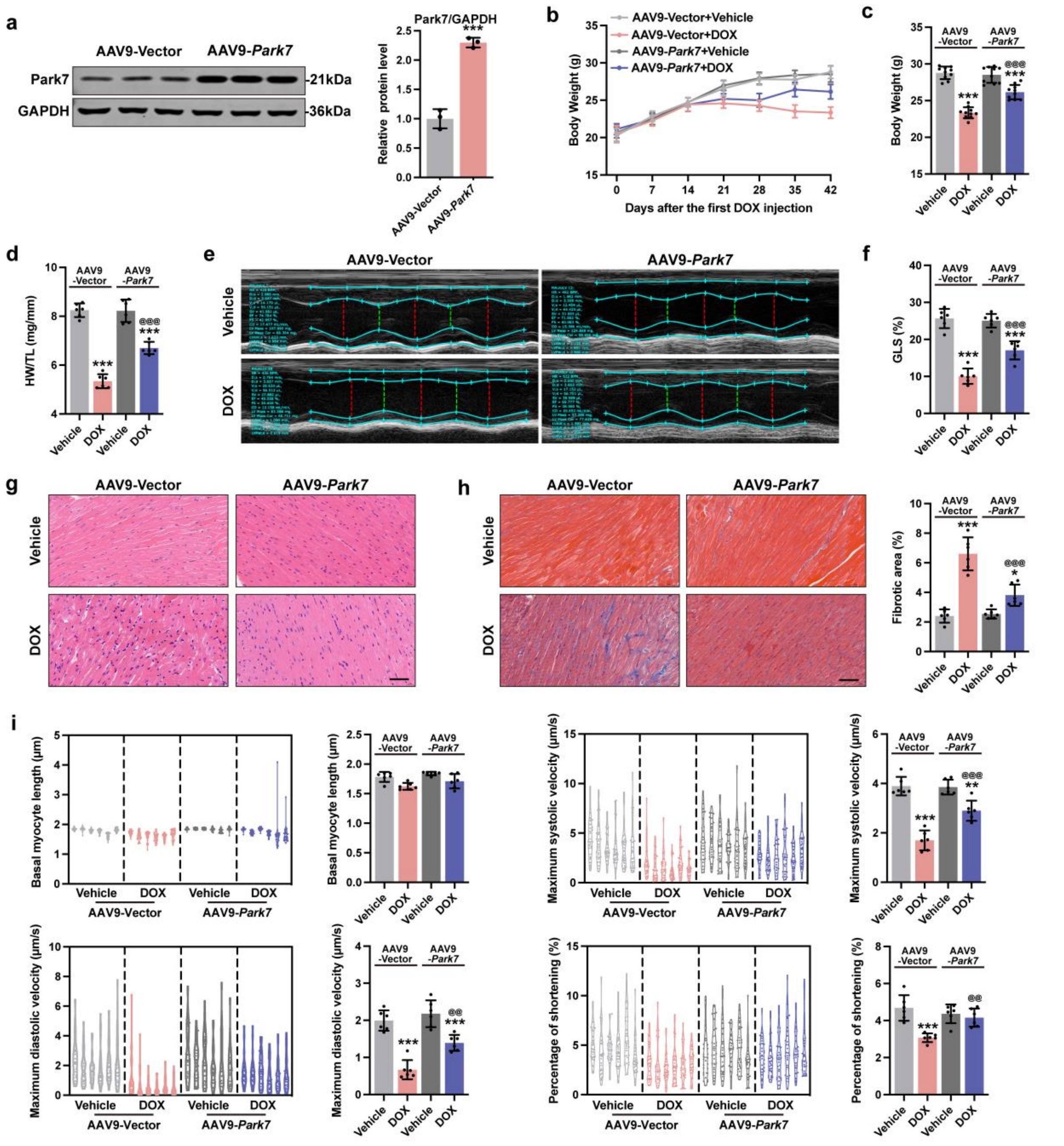


**Figure S9 Overexpression of *Park7* ameliorates DoIC in mice.**

**a)** Representative immunoblots and quantitative analysis of Park7 protein level in AMCMs (n = 3). **b)** Body weight tracing during DoIC model (n = 10). **c)** Body weight of mice before sacrificed (n = 10). **d)** Ratio of heart weight to tibia length (HW/TL; n = 6). **e)** Representative M-mode images of transthoracic echocardiography. **f)** Quantification of global longitudinal strain (GLS; n = 6). **g)** Representative micrographs of H&E staining in heart sections (scale

bars, 50 μm). **h)** Representative micrographs of Masson's trichrome staining and percentage of fibrotic area in heart sections (scale bars, 50 μm; n = 6). **i)** Calculation of basal myocyte length, maximum systolic velocity, maximum diastolic velocity and percentage of shortening of AMCMs (n = 30 AMCMs per mice, 6mice per group). The data are expressed as mean ± SD and analyzed using one-way ANOVA followed by Tukey's post hoc test, *$p < 0.05$, **$p < 0.01$ and ***$p < 0.001$ vs. Vehicle group; @@$p < 0.01$ and @@@$p < 0.001$ vs. AAV9-Vector+DOX group.

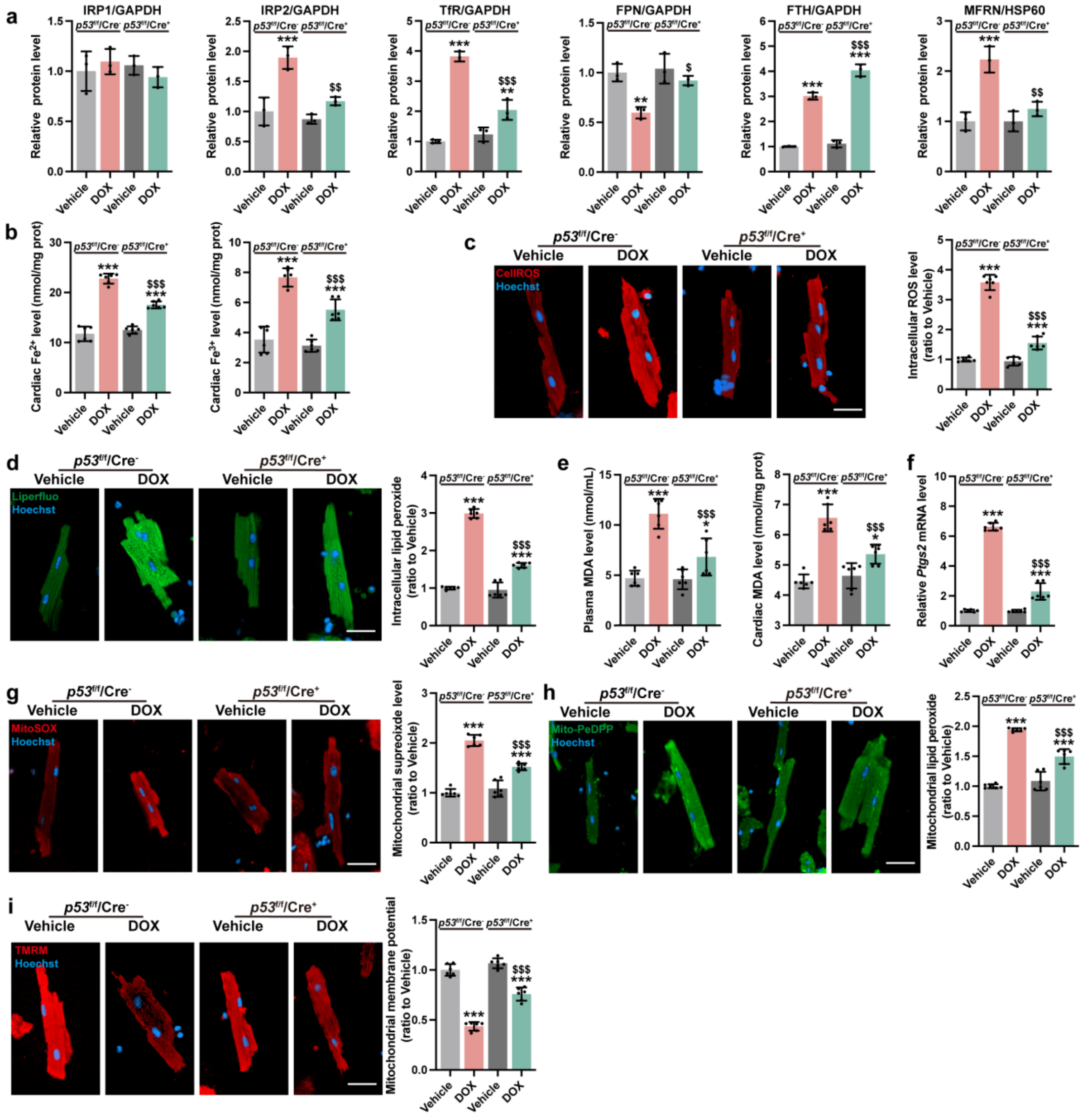


**Figure S10 Knockout of *p53* alleviates DOX induced myocardial iron homeostasis dysfunction and ferroptosis in mice.**

**a)** Quantitative analysis of cellular and mitochondrial iron homeostasis-related proteins in AMCMs in Figure 8e (n = 3). **b)** $Fe^{2+}$ and $Fe^{3+}$ levels in heart tissues (n = 6). **c)** Representative micrographs and quantification of intracellular ROS (CellROS staining) in AMCMs (scale bars, 20μm; n = 6). **d)** Representative micrographs and quantification of

intracellular lipid peroxide (Liperfluo staining) in AMCMs (scale bars, 20µm; n = 6). **e)** MDA level in plasma and heart tissues (n = 6). **f)** *Ptgs2* mRNA expression level in AMCMs (n = 6). **g)** Representative micrographs and quantification of mitochondrial superoxide level (MitoSOX staining) in AMCMs (scale bars, 20µm; n = 6). **h)** Representative micrographs and quantification of mitochondrial lipid peroxide (Mito-PeDPP staining) in AMCMs (scale bars, 20µm; n = 6). **i)** Representative micrographs and quantification of mitochondrial membrane potential (TMRM staining) in AMCMs (scale bars, 20µm; n = 6). The data are expressed as mean ± SD and analyzed using one-way ANOVA followed by Tukey's post hoc test, *p < 0.05, **p < 0.01 and ***p < 0.001 vs. Vehicle group; $p < 0.05, $$p < 0.01 and $$$p < 0.001 vs. *p53*$^{f/f}$/Cre$^{-}$+DOX group.

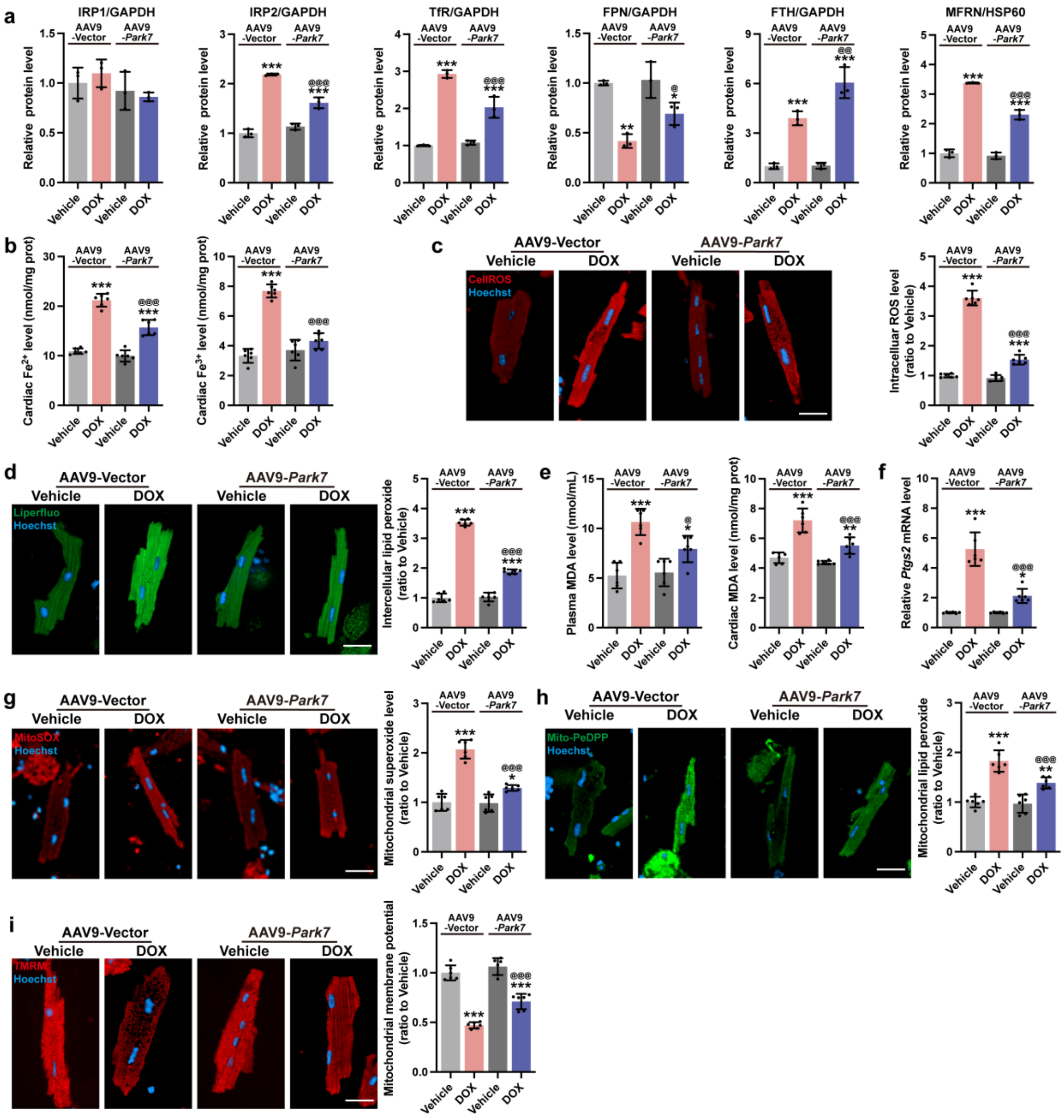


**Figure S11 Overexpression of *Park7* alleviates DOX induced myocardial iron homeostasis dysfunction and ferroptosis in mice.**

**a)** Quantitative analysis of cellular and mitochondrial iron homeostasis-related proteins in AMCMs in Figure 8E (n = 3). **b)** $Fe^{2+}$ and $Fe^{3+}$ levels in heart tissues (n = 6). **c)** Representative micrographs and quantification of intracellular ROS (CellROS staining) in AMCMs (scale bars, 20μm; n = 6). **d)** Representative micrographs and quantification of

intracellular lipid peroxide (Liperfluo staining) in AMCMs (scale bars, 20μm; n = 6). **e)** MDA level in plasma and heart tissues (n = 6). **f)** *Ptgs2* mRNA expression level in AMCMs (n = 6). **g)** Representative micrographs and quantification of mitochondrial superoxide level (MitoSOX staining) in AMCMs (scale bars, 20μm; n = 6). **h)** Representative micrographs and quantification of mitochondrial lipid peroxide (Mito-PeDPP staining) in AMCMs (scale bars, 20μm; n = 6). **i)** Representative micrographs and quantification of mitochondrial membrane potential (TMRM staining) in AMCMs (scale bars, 20μm; n = 6). The data are expressed as mean ± SD and analyzed using one-way ANOVA followed by Tukey's post hoc test, *p < 0.05, **p < 0.01 and ***p < 0.001 vs. Vehicle group; @p < 0.05, @@p < 0.01 and @@@p < 0.001 vs. AAV9-Vector+DOX group.
